# Novel 3-Methyl-1,6-Diazaphenothiazine as an Anticancer Agent—Synthesis, Structure, and In Vitro Anticancer Evaluation

**DOI:** 10.3390/molecules30132779

**Published:** 2025-06-27

**Authors:** Beata Morak-Młodawska, Emilia Martula, Małgorzata Jeleń, Artur Beberok, Zuzanna Rzepka, Sebastian Musiał, Szymon Małek, Marta Karkoszka-Stanowska, Dorota Wrześniok

**Affiliations:** 1 Department of Organic Chemistry, Faculty of Pharmaceutical Sciences in Sosnowiec, Medical University of Silesia, Jagiellońska 4, 41-200 Sosnowiec, Poland; d201074@365.sum.edu.pl (E.M.); manowak@sum.edu.pl (M.J.); 2 Department of Pharmaceutical Chemistry, Faculty of Pharmaceutical Sciences in Sosnowiec, Medical University of Silesia, Jagiellońska 4, 41-200 Sosnowiec, Poland; zrzepka@sum.edu.pl (Z.R.); s85001@365.sum.edu.pl (S.M.); s84920@365.sum.edu.pl (S.M.); marta.karkoszka@sum.edu.pl (M.K.-S.); dwrzesniok@sum.edu.pl (D.W.)

**Keywords:** pyridine derivatives, dipyridothiazines, cytotoxicity, melanoma, apoptosis detection

## Abstract

Pyridine derivatives are widely distributed in nature and have valuable pharmacological properties. The pyridine core can be found in drugs such as sorafenib, zapiclone or prothipendyl. Dipyridothiazines are derivatives of phenothiazines that exhibit valuable anticancer, antioxidant and immunomodulatory activities. In this study, we present the synthesis and preliminary in vitro analysis of anticancer activity towards melanotic (COLO829, G361) and amelanotic (A375, C32) melanoma cells and normal human fibroblasts (HDF) of a series of new tricyclic diazaphenothiazines containing a pyridine scaffold in their structure. The structures of these new molecules was confirmed using spectral techniques, including ^1^H NMR, ^13^C NMR, 2D NMR and HRMS. An in vitro panel of experiments was assessed using the WST-1 assay and cytometric techniques. The two most promising compounds were analyzed for their effect on intracellular GSH levels, mitochondrial membrane potential and their ability to initiate DNA fragmentation to determine the potential mechanism of both cytotoxic and proapoptotic activity. The conducted studies confirmed the ability of the new 3-methyl-1,6-diazaphenothiazines to induce apoptosis in cancer cells, especially in terms of inducing initial as well as late-phase apoptosis. Moreover, the studied compounds were found to induce redox imbalance (evidenced by GSH depletion) in the analyzed melanoma cells, which may be an important factor that directs melanoma cells towards cell death signaling pathways.

## 1. Introduction

Cancer is one of the leading causes of human death worldwide. Among the constantly developing methods of treatment, surgery, radiotherapy, chemotherapy and biotherapy are at the forefront [1,2]. However, this does not change the fact that the effectiveness of treatment is insufficient. Chemotherapeutic agents can cure, prolong life, and reduce tumor burden in order to alleviate symptoms [3]. At the turn of the last two decades, there have been increased efforts by scientists around the world to develop new, selective, safe and more effective chemotherapeutics. Synthetic and natural bioactive compounds with heterocyclic rings play an important role in the development of new scaffolds in drug design and medicinal chemistry [4,5,6,7,8,9]. Among many different heterocyclic systems, one of the most biologically active is the six-membered pyridine ring. Pyridine is a building block of many pharmacologically active substances, including nicotine, vitamin PP, isoniazid or drugs such as sorafenib and zapiclone [10,11,12,13,14,15,16]. The pyridine ring can also be found in tricyclic aza- and diazaphenothiazines, which are pyridobenzothiazines and dipyridothiazines. Prothipendyl is an example of 1-azaphenothiazine, which has moderate neuroleptic properties and, despite being discovered in the 1960s, is still used in medicine today [17]. Additionally, it exhibits antiviral [18] and antitumor [19] activities. Dipyridothiazines, which are modified phenothiazines, possess wide immunomodulatory, antioxidant and antitumor potential [20,21]. The anticancer activity present in this group of compounds is particularly noteworthy. The significant cytotoxic activity of these derivatives, which is stronger than cisplatin, has been documented in relation to cancers including lung cancers HOP-62 and HOP-92, colon cancers COLO 205, HCT-116 and SW-948, renal cancers RXF393 and A498, and leukemia HL-60(TB) and L-1210. Additionally, compounds in this group did not show cytotoxicity towards normal cells within the range of tested concentrations. Studies of the mechanism of anticancer activity of these derivatives have revealed the activation of the mitochondrial apoptosis pathway. Gene expression analysis has confirmed an imbalance among BCL-2 and BAX proteins and the possibility of DNA intercalation [22,23,24,25]. Taking the above information into account, the aim of this study was to effectively synthesize a new 10*H*-3-methyl-1,6-diazphenothiazine and transform it into selected 10-substituted derivatives. The key challenge of our research was to document the structure of these compounds and determine their anticancer potency against selected melanoma cancer cell lines, as well as normal skin cells, to analyze the mechanism of anticancer activity.

## 2. Results and Discussion

### 2.1. Chemistry Part

#### Synthesis and Structure Analysis

**Scheme 1:**
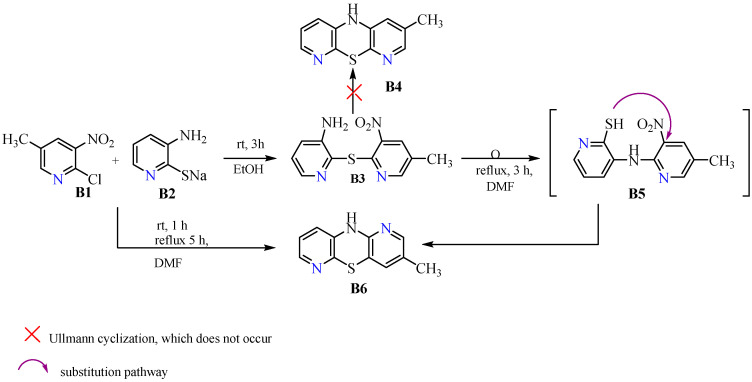
Synthetic routes to 10*H*- and 10-substituted 3-methyl-1,6-diazaphenothiazines (**B6**).

Phenothiazines are fully synthetic compounds obtained mainly in reactions involving S→N-type Smiles rearrangements, using various substrates and conditions. According to reports in the literature, such rearrangements are performed by *ortho*-aminodiphenylsulfide or azinylsulfide with a leaving group, which can be a nitro group. An amine is then formed, often unstable, which undergoes rapid C-N cyclization, forming a 1,4-thiazine ring, and thus a phenothiazine or azaphenothiazine [26,27]. Most often, this type of rearrangement occurs in alkaline environments and occurs less often in neutral or acidic environments. A competing reaction with this type of rearrangement is Ullmann cyclization, which leads to the formation of isomeric molecules [27,28]. Our synthesis, reported in Scheme 1, of the new diazaphenothiazine was initiated by obtaining 3-amino-3′-nitro-2,4′-dipyridinyl sulfide (**B3**). We obtained this compound via efficient synthesis of 2-chloro-3-nitro-5-methylpyridine (**B1**) and sodium 3-amino-2-pyridinethiolate (**B2**) in ethanol, at room temperature. Then, using the above sulfide, we performed the reaction in boiling DMF. The key step in this reaction was the S→N Smiles rearrangement which formed 2,4′-dipyridylamine (**B5**), followed by thiazine ring closure via intramolecular nucleophilic substitution of the nitro group by a thiolate group, thus forming the central 1,4-thiazine ring which is the core structure of the intended 10*H*-3-methyl-1,6-diazaphenothiazine (**B6**). The above-mentioned amine (**B5**) was not isolated during the reaction, and that is why it was placed in brackets in Scheme 1. In this process, the final diazaphenothiazine (**B6**) was obtained with a high yield (79%).

10*H*-3-methyl-1,6-diazaphenothiazine (**B6**) was also obtained directly with a lower yield of 62% in a reaction using substrates (**B1**) and (**B2**), which were first mixed together in stoichiometric amounts and then heated in boiling DMF. Analyzing the mechanism of this reaction, it can be assumed that both pyridine derivatives must have formed 3-amino-3′-nitro-2,4′-dipyridinyl sulfide (**B3**) in the first step, at room temperature, which then underwent the Smiles rearrangement described earlier. The reduced efficiency of this process may be due to difficulties associated with eliminating the leaving group that must occur during this process. A possible problem that could have occurred in the above process was Ullmann cyclization leading to the formation of isomeric dipyridothiazine (**B4**). However, this route of synthesis was excluded on the basis of structural studies, specifically 2D NMR analysis of the final product (**B6**). The synthetic routes are presented in Scheme 1.

**Scheme 2:**
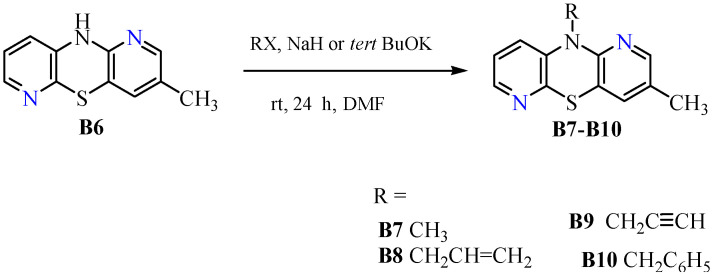
Synthetic routes to 10-substituted 3-methyl-1,6-diazaphenothiazines (**B7**–**B10**).

In a further stage of the synthesis, the parent dipyridothiazine (**B6**) was transformed into 10-substituted derivatives bearing methyl (**B7**), allyl (**B8**), propargyl (**B9**) and benzyl (**B10**) substituents on the thiazine nitrogen atom (in the central 1,4-thiazine ring). These reactions were performed using the appropriate alkyl halides, in an inert solvent such as DMF, in the presence of a strong base such as NaH or potassium *tert*-butoxide, at room temperature. All reactions, reported in Scheme 2, were carried out in high yields.

The obtained 10-substituted derivatives were in the form of solids (**B7**, **B9**) or oils (**B8**, **B10**) and were stable compounds. The progress of the reactions was monitored using thin layer chromatography (TLC), the chromatograms of which confirmed the characteristic color changes for azaphenothiazines from light yellow to yellow celadon in the 364 nm range.

**Figure 1:**
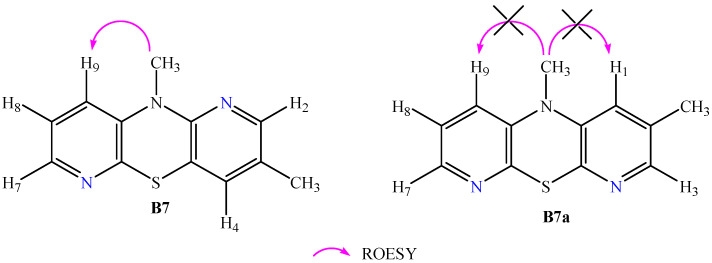
Interaction of the methyl group protons located on thiazine nitrogen observed in product **B7** and the hypothetical product **B7a**, which was not formed in the reactions carried out.

As mentioned earlier, in the synthesis of phenothiazines via the Smiles rearrangement, a competing reaction may occur in the form of Ullmann cyclization leading to the formation of isomeric dipyridothiazine [27,28]. This fact provided the rationale for performing an in-depth structural analysis to confirm the structure of our dipyridothiazine (**B6**). HRMS revealed the molecular formula C_11_H_9_N_3_S, which is analogous to both the product (**B6**) and the hypothetical product (**B4**). Evidence for the structure of dipyridothiazine (**B6**) was provided by 2D NMR spectra. In order to find the position of azine nitrogen atoms in the dipyridothiazine system, our product (**B6**) was methylated with methyl iodide in dry DMF in the presence of sodium hydride. In this way, derivative (**B7**) was obtained, for which a simple ^1^H NMR spectrum was performed, revealing seven signals: two methyl groups and five protons of the dipyridothiazine system. To determine the location of nitrogen atoms in the dipyridothiazine core, a 2D NMR spectrum (ROESY—*Rotating-frame nuclear Overhauser Effect Correlation Spectroscopy*) was performed, enabling observation of the interactions of individual protons in space [29]. Irradiation of the second methyl protons at 3.45 ppm revealed an interaction with only one multiplet proton, located at 7.02 ppm, which was assigned as proton H_9_. This indicated that the nitrogen atom of the dipyridothiazine system must be located at position one of the entire dipyridothiazine core. If the studied compound was an analogous methyl derivative designated as **B7a** built on dipyridothiazine core **B4**, interactions with two protons would be observed in the ROESY spectrum, one of which would be a singlet and the other a multiplet (Figure 1). However, this was not observed.

In the next stage of structural analysis, after assigning chemical shifts to all protons, ^13^C NMR spectra and short- and long-range correlation spectra of ^1^H-^13^C NMR HSQC (*Heteronuclear Single Quantum Coherence*) and HMBC (*Heteronuclear Multiple Bond Correlation*) were recorded. This enabled the assignment of individual signals to specific carbon atoms. The complete assignment of proton and carbon signals contained in the 2D NMR spectra showing both ^1^H-^1^H (COSY) and ^1^H-^13^C (HSQC and HMBC) interactions is provided in the Supplementary Materials. Spectroscopic analysis confirmed that the methylated product was 3,10-dimethyl-1,6-diazaphenothiazine (**B7**). This result also confirmed that the conducted reaction of the parent dipyridothiazine synthesis proceeded via the **S**→**N** Smiles rearrangement and excluded the Ullmann cyclization process.

### 2.2. In Vitro Anticancer Activity

#### 2.2.1. Viability Assay

**Figure 2:**
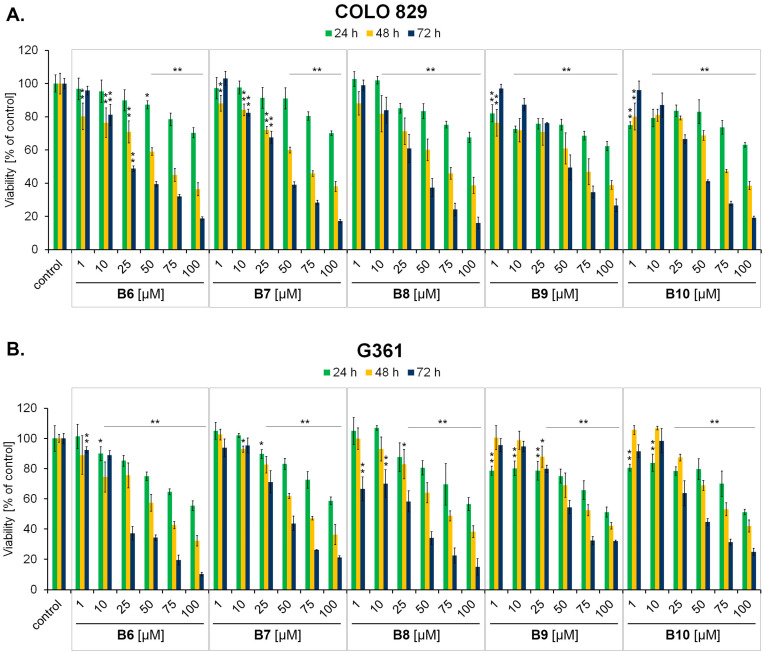
The effect of 3-methyl-1,6-diazaphenothiazine derivatives **B6**–**B10** on melanotic COLO829 (**A**) and G361 (**B**) melanoma cell viability after 24 h, 48 h and 72 h treatments. The results were expressed as a percentage of the control. Bars represent the mean ± SD of three independent experiments, * *p* < 0.05 and ** *p* < 0.005 vs. relative control.

**Figure 3:**
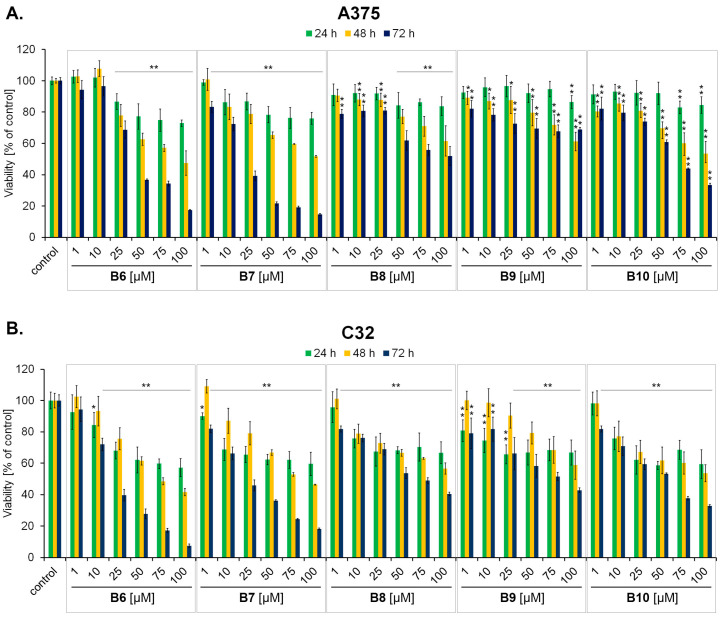
The effect of 3-methyl-1,6-diazaphenothiazine derivatives **B6–B10** on amelanotic A375 (**A**) and C32 (**B**) melanoma cell viability after 24 h, 48 h and 72 h treatments. The results were expressed as a percentage of the control. Bars represent the mean ± SD of three independent experiments, * *p* < 0.05 and ** *p* < 0.005 vs. relative control.

In the first part of the in vitro panel of experiments, the cytotoxicity of the studied compounds was assessed. The WST-1 assay demonstrated that compounds **B6**–**B10** exert high cytotoxic potential, expressed as a decrease in cell viability, in both melanotic (COLO829, G361) (Figure 2) and amelanotic (A375, C32) (Figure 3) melanoma cells. All the studied compounds affected the viability of all tested melanoma cell lines in both a concentration- and time-dependent manner. Taking into account the estimated IC_50_ parameter values—the concentration that reduced the viability of cells by 50% when compared to the control (Table 1)—the strongest cytotoxic activity was detected using a 72 h exposure time for the compound **B7** in the case of A375 cells (IC_50_ = 17.64 µM), **B6** and **B7** in the case of C32 cells (IC_50_ = 18.14 µM and 22.23 µM, respectively), **B6** and **B8** in the case of COLO829 cells (IC_50_ = 30.15 µM and 31.44 µM, respectively) and **B6** and **B8** in the case of G361 (IC_50_ = 22.85 µM and 24.57 µM, respectively). Respondek et al. [30] have demonstrated that the use of dacarbazine only in the highest analyzed concentration (50 µM) with a 72 h incubation time caused the maximum reduction in COLO829 cell viability—by about 25%. In the case of C32 cells under the same exposure conditions, the analyzed parameter was found to be about 50% [31]. When analyzing the obtained values of IC_50_ parameters for the studied compounds (**B6**–**B10**), it may be concluded that the novel 3-methyl-1,6-diazaphenothiazine derivatives possess higher cytotoxic activity than dacarbazine—the standard chemotherapeutic drug used in malignant melanoma treatment.

**Figure 4:**
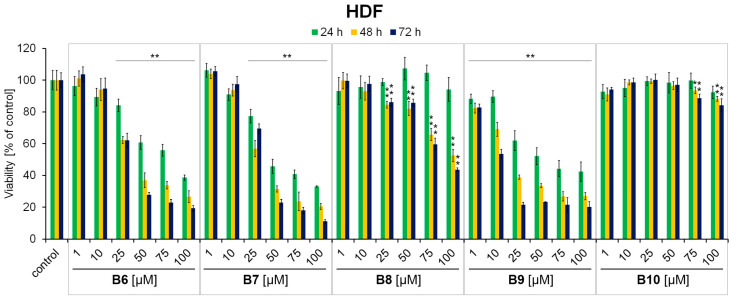
The effect of 3-methyl-1,6-diazaphenothiazine derivatives **B6**–**B10** on normal human fibroblast (HDF) viability after 24 h, 48 h and 72 h treatments. The results were expressed as a percentage of the control. Bars represent the mean ± SD of three independent experiments, * *p* < 0.05 and ** *p* < 0.005 vs. control.

Screening analysis using normal human fibroblasts (HDF) revealed that only compound **B8** and **B10** exerted high selectivity in the mode of action (Figure 4). Significant viability reduction (of >20% in comparison to the control) was detected only in the case of compound **B8** in concentrations of 75 µM, 100 µM, following 48 h and 72 h of incubation. In the case of compound **B10**, the viability of cells did not decrease by more than 10% at any measurement point. In contrast, compounds **B6**, **B7** and **B9** significantly decreased the value of the analyzed parameter (reduction in cell viability by more than 20% in comparison to the control) at all the studied incubation time points for concentrations equal to and above 25 µM. Therefore 3-methyl-1,6-diazaphenothiazine derivatives **B8** and **B10** were selected for further analysis. Moreover, based on the conducted analysis, amelanotic C32 and melanotic G361melanoma cells were used as an experimental model for the subsequent intracellular GSH assessment and apoptosis induction panel of experiments.

In our previous studies, novel diazaphenothiazines with 1,2,3-triazole substituents were found to exert a broad spectrum of anticancer activity, and the compound containing the *p*-chlorobenzyl substituent possessing low cytotoxicity against normal human fibroblasts was the most promising [22]. In the next case of tetracyclic azaphenothiazines with the quinolone ring, the seven most active analogues were demonstrated to possess high cytotoxic activity and the ability to induce apoptosis [32]. Thus, it may be concluded that the diazaphenothiazine ring could serve as the core structure for novel anticancer agents with (i) strong cytotoxic activity against cancer cells and (ii) low impact on normal cells.

#### 2.2.2. Vitality Assay—GSH Level Assessment

**Figure 5:**
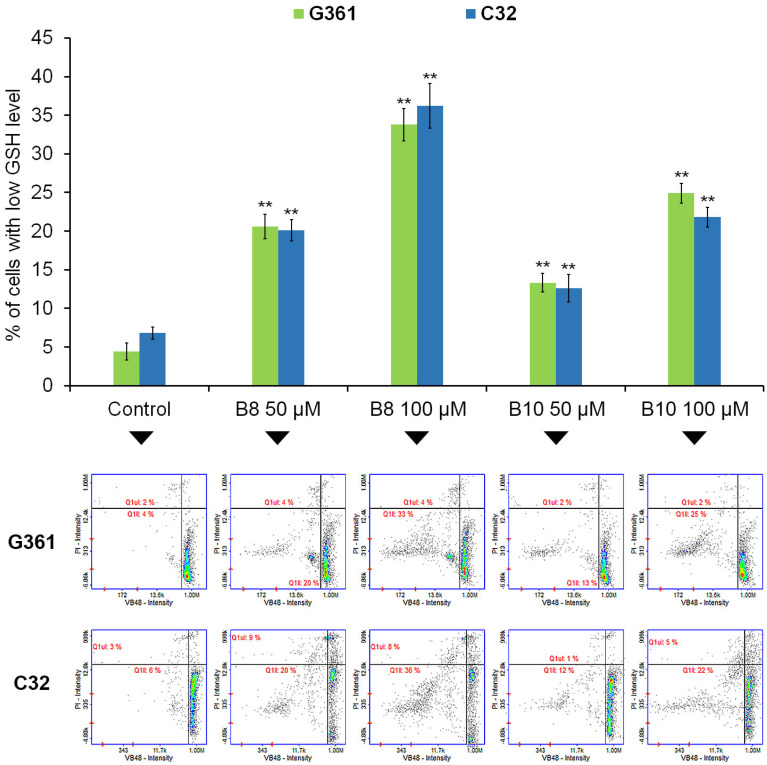
The effect of 3-methyl-1,6-diazaphenothiazine derivatives **B8** and **B10** on intracellular GSH levels in G361 melanotic and C32 amelanotic melanoma cells. Cells were treated with the studied compounds in concentrations of 50 µM and 100 µM for 72 h. Graphs represent the mean ± SD of three independent experiments completed in triplicate from the vitality assay assessing GSH levels. ** p < 0.005 vs. control. Representative histograms for three independent experiments with similar results, where Q1ll represents GSH-depleted cells; Q1ul represents dead cells.

Glutathione (GSH) constitutes an important component of the cellular antioxidative system. In cancer cells, a high level of GSH is essential to scavenge excessive reactive oxygen species (ROS) levels which makes it a potential target for cancer therapy. In addition, loss of intracellular GSH makes cancer cells more susceptible to oxidative stress and chemotherapeutic agents. Moreover, GSH depletion has been proven to improve the therapeutic efficacy of ROS-based therapy (e.g., photodynamic therapy, sonodynamic therapy and chemodynamic therapy, ferroptosis, and chemotherapy) [33,34,35,36]. In the current study, we examined whether compounds **B8** and **B10**, which possess both (i) high cytotoxic potential and (ii) selectivity in the mode of action, affect the cellular GSH level in C32 amelanotic and G361 melanotic melanoma cells (Figure 5). We found that the exposure of cells to both the studied compounds led to a significant increase in the population of cells with low GSH levels. In the case of G361 melanoma cells, the value of the analyzed parameter increased from about 4% (control) to 21% (**B8** 50 µM), 33% (**B8** 100 µM), 13% (**B10** 50 µM), and 25% (**B10** 100µM). For C32 melanoma cells, the percentage of cells with low intracellular GSH levels increased from about 6% (control) to 20% (**B8** 50 µM), 36% (**B8** 100 µM), 12% (**B10** 50 µM), and 22% (**B10** 100µM). In a previously published study, it was demonstrated that the observed GSH depletion after lomefloxacin (fluoroquinolone antibiotic) and UVA radiation treatment of COLO829 and C32 melanoma cells was associated with a simultaneous increase in reactive oxygen species (ROS) generation when assessed with the use of DCFDA assay [37]. Analyzing the obtained data from the current assessment, it can be observed that compounds **B8** and **B10** exerted a high capacity to induce redox imbalance—evidenced by GSH depletion as a potential consequence of excessive ROS generation in the studied cells. This represents an important factor that directs melanoma cells towards cell death signaling pathways.

#### 2.2.3. Apoptosis Detection Assay

The latest scientific literature indicates that the mainstay and goal of clinical oncology has been the development of therapies promoting the effective elimination of cancer cells by apoptosis. Moreover, the antitumor efficacy of several FDA-approved agents targeting cell survival and proliferation pathways in cancer cells is also dependent on their effects on apoptosis signaling pathways [38,39,40]. Encouraged by the findings that compounds **B8** and **B10** affect melanoma cell viability and reduce intracellular GSH levels, in the next part of analysis, we examined the capacity of the tested derivatives to induce apoptosis by analyzing mitochondrial membrane potential and DNA fragmentation in the studied in vitro experimental model.

**Figure 6:**
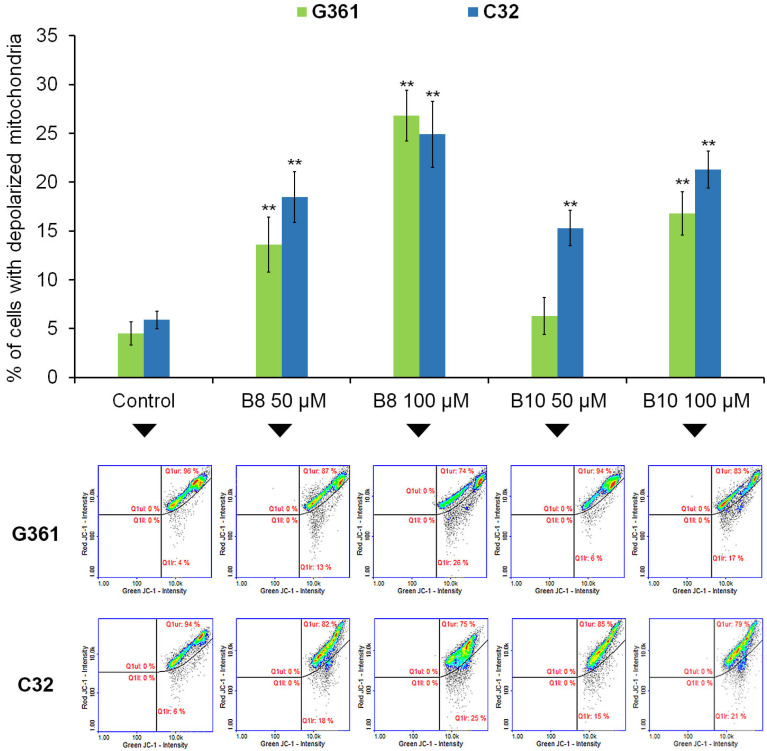
The effect of **B8** and **B10** diazaphenothiazine derivatives on mitochondrial membrane potential in G361 melanotic and C32 amelanotic melanoma cells. Cells were treated with the studied compounds in concentrations 50 µM and 100 µM for 72 h. Graphs represent the mean ± SD of three independent experiments completed in triplicate from the mitochondrial potential assay. ** p < 0.005 vs. control. Representative histograms for three independent experiments with similar results, where Q1ur represents healthy cells; Q1lr represents depolarized cells.

Mitochondria are essential organelles whose primary function is to convert nutrients into energy, which is subsequently exported and used throughout cells. The outer mitochondrial membrane is permeable to molecules, which enables the exchange of respiratory chain substrates and products between mitochondria and the cytosol. The inner mitochondrial membrane is highly impermeable, an essential characteristic for generating the electrochemical potential necessary for oxidative phosphorylation and ATP production [41]. Mitochondria also have an important role in triggering and regulating apoptosis where the regulation of the inner mitochondrial membrane permeability appears to be critical in this process [42,43]. In the current study, we revealed that the studied 3-methyl-1,6-diazaphenothiazine derivatives increase the number of depolarized (early apoptotic) melanoma cells (Figure 6). In the case of melanotic G361 cells, the value of the analyzed parameter increased from about 4% (control) to 13% (**B8** 50 µM), 26% (**B8** 100 µM), 6% (**B10** 50 µM), and 17% (**B10** 100 µM). Interestingly, a similar pattern was observed in amelanotic C32 cells, where the number of cells with decreased mitochondrial membrane potential increased from about 6% (control) to 18% (**B8** 50 µM), 25% (**B8** 100 µM), 15% (**B10** 50 µM), and 21% (**B10** 100 µM).

**Figure 7:**
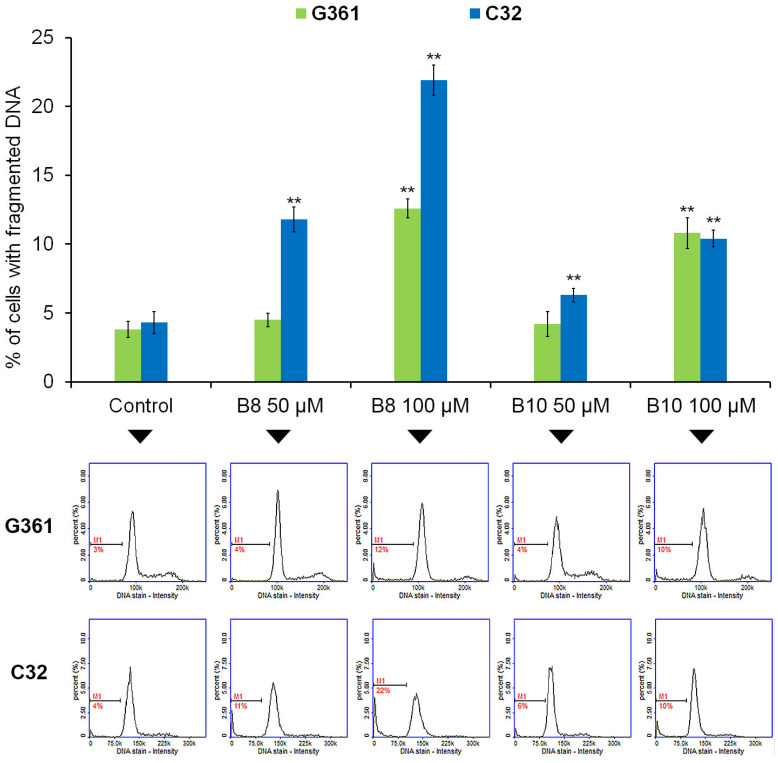
The effect of compounds **B8** and **B10** on DNA fragmentation in G361 melanotic and C32 amelanotic melanoma cells. Cells were treated with the studied compounds in concentrations 50 µM and 100 µM for 72 h. Graphs represent the mean ± SD of three independent experiments completed in triplicate from the DNA fragmentation assay. ** p < 0.005 vs. control. Representative histograms for three independent experiments with similar results, where M1 represents percentages of cells with fragmented DNA.

In the last part of the in vitro anticancer panel of experiments, we assessed the ability of the tested compounds to induce DNA fragmentation—the late phase of apoptosis. When analyzing the melanotic G361 melanoma model, a significant increase in the analyzed parameter was observed for the compounds **B8** (an increase from about 3% to 12%) and **B10** (an increase from about 3% to 10%) only in the highest assessed concentration (100 µM) (Figure 7). In the case of C32 melanoma cells, the increase in the percentage of cells with fragmented DNA was observed for the **B8** derivative in concentrations of 50 µM and 100 µM—an increase from about 4% to 11% and 22%, respectively. There was also an increase from about 4% to 10% for the **B10** derivative at a concentration of 100 µM. Taking into account the data obtained from the apoptosis induction assays, it was revealed that the analyzed dipyridothiazines (**B8** and **B10**) possess a strong ability to initiate apoptosis via an intrinsic signaling pathway. Moreover, the proapoptotic activity of the **B8** and **B10** derivatives was confirmed by their capacity to trigger DNA fragmentation in melanotic and amelanotic melanoma cells. Previously, it was demonstrated that novel diazaphenothiazines with 1,2,3-triazole substituents affect the gene expression ratio of *BCL-2*/*BAX* in MDA-MB231 breast cancer cells and activate mitochondrial apoptosis as the internal pathway of cell death. Moreover, the transcriptional activity of these genes in SNB-19, Caco-2, and A549 cells suggests a different pathway for cell death, possibly associated with the external pathway of apoptosis [22]. It can therefore be assumed that the 3-methyl-1,6-diazaphenothiazine derivatives analyzed in the current study may modulate the expression of *BCL-2* and *BAX* genes as a molecular targets underlying their proapoptotic activity.

## 3. Materials and Methods

### 3.1. Chemistry Part

Melting points of the new compounds were determined in open capillaries using a Boetius melting point apparatus (Stuart Equipment, Stone, UK) [43,44] and were uncorrected. ^1^H NMR, COSY, ROESY, HSQC, HMBC spectra were recorded on a Bruker AscendTM 600 spectrometer at 600 MHz (Bruker, Rheinstetten, Germany) in deuterochloroform. ^13^C NMR spectra were recorded at 150 MHz on a Bruker AscendTM 600 spectrometer [45]. The HR MS spectra (ESI—Electro Spray Ionisation) were run on a Brucker Impact II (Bruker, Billerica, MA, USA). Thin layer chromatography was performed according to the described methodology [46] on aluminum oxide 60 F254 neutral (type E) (Merck 1.05581, San Jose, CA, USA) using chloroform as the eluent.

The following commercial reagents and solvents were used to perform the syntheses in the experimental studies: 2-chloro-3-nitro-5-methylpyridine (Sigma-Aldrich, Burlington, MA, USA), DMF, ethanol, chloroform (POCh, Gliwice, Poland). Sodium 3-amino-2-pyridinothiolate (**B2**) was obtained according to reference [21].

*Synthesis of 3-amino-3′-nitro-5′-methyl-2,2-dipyridinyl sulfide* (**B3**)

A total of 158 mg (1 mmol) of 2-chloro-3-nitro-5-methylpyridine (**B1**) was added to a solution of sodium 3-amino-2-pyridinethiolate (**B2**) (148 mg, 1 mmol) in dry ethanol (10 mL). The mixture was stirred at room temperature for 3 h and then, the resulting brown crystals were filtered off, washed with ethanol and air dried to obtain 3-amino-3′-nitro-5′-methyl-2,2-dipyridinyl sulfide (**B3**). 

The dry product was recrystallized from ethanol yielding 200 mg (78%), m.p. 110–111 °C.

^1^H NMR (CDCl_3_) δ: 2.37 (s, 3H, CH_3_), 4.28 (broad s, 2H, NH_2_) 7.12 (m 1H), 7.20 (m, 1H), 8.11 (m, 1H), 8.31 (s, 1H,), 8.34 (s, 1H). ^13^C NMR 17.33, 122.52, 125.68, 130.40, 133.78, 134.44, 135.96, 140.65, 146.78, 153.00, 154.40.

HRMS (EI) *m*/*z* for [C_11_H_10_N_4_O_2_S + H], calc. 263.0603, found: 263.0627.

TLC: *R_f_* = 0.35 (Al_2_O_3_/CHCl_3_)

*Synthesis of 10H-3-methyl-1,6-diazaphenothiazine* (**B6**)

A total of 158 mg, (1 mmol) of 2-chloro-3-nitro-5-methylpyridine (**B1**) was added to a solution of sodium 3-amino-4-pyridinethiolate (**B2**) (148 mg, 1 mmol) in dry DMF (10 mL). The mixture was stirred at room temperature for 1 h and was then refluxed for 5 h. After cooling, the reaction mixture was evaporated in vacuo. The dry residue was dissolved in CHCl_3_ and purified using column chromatography (Al_2_O_3_, CHCl_3_) to obtain 10*H*-3-methyl-1,6-diazaphenothiazine (**B6**) (140 mg, 67.4%), m.p. 149–150 °C.

*Synthesis of 10H-3-methyl-1,6-diazaphenothiazine* (**B6***) in cyclization of 3-amino-3′-nitro-5′-methyl-2,2-dipyridinyl sulfide* (**B3**)

A total of 230 mg (1 mmol) of 3-amino-3′-nitro-5′-methyl-2,2′-dipyridyl (**B3**) and 5 mL of anhydrous DMF was added to a round-bottomed flask. The flask was heated for 3 h at reflux. Then, the solvent was evaporated on a vacuum evaporator, and the remaining oil was dissolved in chloroform and purified using a chromatographic column (Al_2_O_3_, chloroform-ethanol 10:1, *v*/*v*). A total of 170 mg (79%) of the compound was obtained, which was identified as 10*H*-3-methyl-1,6-diazaphenothiazine (**B6**) with a melting point of 149–150 °C.

^1^H NMR (CDCl_3_) δ: 2.15 (s, 3H, CH_3_), 6.70 (dd, J = 7.8 Hz, J = 1.2 Hz 1H, H_9_), 6.85 (dd, J = 7.2 Hz, J = 3 Hz, 1H, H_8_), 7.02 (s, 1H, H_4_), 7.54 (broad s, 1H, NH), 7.57 (s, 1H, H_2_), 7.88 (dd, J = 4.8 Hz, J = 1.2 Hz, 1H, H_7_). ^13^C NMR 17.38, 114.89, 120.35, 122.25, 127.77, 135.44, 141.04, 143.09, 143.48, 148.99, 152.03.

HRMS (EI) *m*/*z* for [C_11_H_9_N_3_S + H], calc. 216.0595, found: 216.0616.

TLC: *R_f_* = 0.25 (Al_2_O_3_/CHCl_3_)

*General procedure for synthesis of 10-substituted 3-methyl-1,6-diazaphenothiazines derivatives* (**B7**, **B8**, **B10**)

NaH (48 mg, 2 mmol, 60% NaH in mineral oil washed out with hexane) was added to a solution of 10*H*-3-methyl-1,6-diazaphenothiazine (**B6**) (230 mg, 1 mmol) in dry DMF (5 mL). The reaction mixture was stirred at room temperature for 1 h and then alkyl halide (methyl iodide, allyl bromide, benzyl chloride, 3 mmol) was added and the stirring was continued for 24 h. The mixture was poured into water (15 mL), extracted with CHCl_3_ and dried using anhydrous Na_2_SO_4_. The obtained product was purified using column chromatography (Al_2_O_3_, CHCl_3_) to give the following:

(a) 3,10-dimethyl-1,6-diazaphenothiazine (**B7**) (214 mg, 93%); m.p. 159–161 °C.
  ^1^H NMR (CDCl_3_) δ: 2.22 (s, 3H, CH_3_), 3.44 (s, 3H, N-CH_3_), 7.02 (d, J = 7.8 Hz, 1H, H9), 7.08 (dd, J = 7.8 Hz, J = 4.2 Hz, 1H, H8), 7.19 (s, 1H, H4), 7.86 (s, 1H, H2), 8.01 (d, J = 4.2 Hz 1H, H7). ^13^C NMR (CDCl_3_) δ: 17.28, 33.29, 116.51, 120.64, 122.34, 127.90, 136.10, 139.94, 141.88, 144.08, 144.37, 150.78.
  HRMS (EI) *m*/*z* for [C_12_H_11_N_3_S + H], calc. 230.0752, found: 230.0713.
  TLC: *R_f_* = 0.62 (Al_2_O_3_/CHCl_3_)
(b) 3-metyhl-10-allyl-1,6-diazaphenothiazine (**B8**) (200 mg, 78%); an oil:
  ^1^H NMR (CDCl_3_) δ: 2.18 (s,3H, CH_3_), 4.61 (m, 2H, N-CH_2_), 5.26 (m, 2H, =CH_2_), 5.98 (m, 1H, CH), 6.98 (m, 2H), 7.09 (s, 1H), 7.79 (s, 1H), 7.92 (m, 1H). ^13^C NMR 17.21, 29.70, 47.75, 115.41, 117.21, 121.63, 122.08, 128.06, 135.44, 141.02, 143.81, 144.74, 149.74, 152.70.
  HRMS (EI) *m*/*z* for [C_14_H_13_N_3_S + H], calc. 256.0908, found: 256.0853.
  TLC: *R_f_* = 0.46 (Al_2_O_3_/CHCl_3_)
(c) 3-methyl-10-benzyl-1,6-diazaphenothiazine (**B10**) (210 mg, 72%); an oil:
  ^1^H NMR (CDCl_3_) δ: 2.20 (s, 3H, CH_3_), 5.27 (s, 2H, CH_2_), 6.73 (m,1H), 6.83 (m, 1H), 7.15 (s, 1H), 7.39 (m, 5H, C_6_H_5_), 7.76 (s, 1H), 7.89 (m, 1H). ^13^C NMR δ: 17.28, 48.92, 115.19, 122.21, 126.45, 127.19, 127.75, 128.41, 128.83, 135.75, 136.05, 140.20, 143.81, 143.94, 145.02, 149.77.
  HRMS (EI) *m*/*z* for [C_18_H_15_N_3_S + H], calc. 306.1065, found: 306.1091.
  TLC: *R_f_* = 0.84 (Al_2_O_3_/CHCl_3_)

*Synthesis of 3-methyl-10-propargyl-3,6-diazaphenothiazines* (**B9**)

Potassium *tert*-butoxide (40 mg, 0.72 mmol) was added to a suspension of the parent compound (**B6**) (107 mg, 0.5 mmol) in dry DMF (10 mL). The mixture was stirred at room temperature for 1 h. Then, an 80% solution of propargyl bromide (0.05 mL, 0.64 mmol) in dry toluene was added dropwise. The solution was stirred at room temperature for 24 h and poured into water (20 mL), extracted with methylene chloride (20 mL), dried with anhydrous Na_2_SO_4_, and evaporated into brown oil. The residue was purified using column chromatography (silica gel, CHCl_3_) to yield 90 mg (70%) of 3-methyl-10-propargyl-1,6-diazaphenothiazine (**B9**), m.p. 139–141 °C.

^1^H NMR (CDCl_3_) δ: 2.19 (c, 3H, CH_3_), 2.32 (s, 1H, C-H), 4.66 (d, J = 2.4 Hz, 2H, CH_2_), 7.04 (m, 1H), 7.13 (s, 1H), 7.26 (m, 1H), 7.87 (s, 1H), 8.0 (m, 1H). ^13^C NMR δ: 17.28, 34.86, 72.37, 79.20, 115.41, 120.75, 121.95, 128.12, 135.35, 142.72, 144.43, 145.06, 149.70, 155.13.

HRMS (EI) *m*/*z* for [C_14_H_11_N_3_S + H], calc. 254.0752, found: 254.0769.

TLC: *R_f_* = 0.80 (Al_2_O_3_/CHCl_3_)

### 3.2. In Vitro Assay

#### 3.2.1. Cell Culture and Treatment

Human A375, C32 amelanotic and COLO829, G361 melanotic melanoma cells, purchased from ATCC (ATCC Manassas, VA, USA), were incubated in a humidified 5% CO_2_ incubator at 37 °C. The cells were cultured in the recommended medium supplemented with inactivated fetal bovine serum and the following antibiotics: penicillin (100 U/mL), neomycin (10 μg/mL) and amphotericin B (0.25 μg/mL). Treatment with the studied compounds (**B6**–**B10**) began 24 h after seeding. The analyzed compound solutions were obtained by diluting stock solutions in a culture medium. The analysis via WST-1 assay was used for compound concentrations ranging from 1 µM to 100 µM. Other studies, concerning the assessment of intracellular GSH levels and apoptosis, were performed on the **B8** and **B10** derivatives at concentrations of 50 μM and 100 µM.

#### 3.2.2. Cell Viability Assessment—WST-1 Assay

The viability of cells was assessed using the cell proliferation reagent WST-1. The reagent is a tetrazolium salt (slightly red) that can be reduced in viable cells to formazan dye (dark red) by mitochondrial dehydrogenases. In the analysis, WST-1 was added to cells cultured in 96-well microplates (10 µL/well) 3 h before the measurement. Absorbance at 440 nm was read using a microplate reader (Infinite 200 PRO, TECAN, Männedorf, Switzerland), and 650 nm was used as the reference wavelength. In the experiment, control samples were normalized to 100%. All the examined samples were calculated as percentages of the control.

#### 3.2.3. Analysis of GSH Level and Apoptosis

All the experiments were performed using the fluorescence image cytometer NucleoCounter^®^ NC-3000™ (ChemoMetec, Lillerød, Denmark). For the vitality assay, treatment cells were stained with solution 5 according to the manufacturer’s protocol and analysis was conducted using NucleoView NC-3000 software 2.1.25.12 (ChemoMetec, Lillerød, Denmark) according to the vitality assay protocol.

The mitochondrial potential assay was conducted using fluorescent cationic dye JC-1, which accumulates in the mitochondria in a potential-dependent manner. After cell treatment, samples were suspended in 12.5 µL of solution 7 (JC-1) (ChemoMetec, Lillerød, Denmark) and then incubated at 37 C for 10 min. In the next step, the MDA-MB-231 cells were centrifuged for 5 min at 400× *g*, washed twice with PBS and resuspended in 0.25 mL of solution 8 (DAPI) (ChemoMetec, Lillerød, Denmark). In the last step of sample preparation, cells were loaded into 8-chamber NC-Slides A8™ and analyzed immediately using the mitochondrial potential assay protocol. Scatterplots of the obtained results were used to demarcate the percentage of cells with a different mitochondrial membrane potential.

For the DNA fragmentation assay, cells were fixed with 70% cold ethanol (24 h at 0–4 °C). In the next step of the analysis, the cell samples were washed with PBS, centrifuged, and stained with solution 3 (ChemoMetec, Lillerød, Denmark) containing DAPI and 0.1% triton X-100 in PBS. The analysis was conducted according to the DNA fragmentation assay protocol.

## 4. Conclusions

In summary, we have presented an effective synthesis of new 3-methyl-1,6-diazaphenothiazines obtained via two synthetic routes: using 3-amino-3′-nitro-5′-methyl-2,2-dipyridinyl sulfide and using pairs of appropriately selected disubstituted pyridine derivatives: 2-chloro-3-nitro-5-methyl-pyridine and sodium 3-amino-2-pyridinethiolate. Spectroscopic studies confirmed that the formation of the new 10*H*-3-methyl-1,6-diazaphenothiazine core (**B6**) proceeded via an **S**→**N** Smiles rearrangement. Advanced ^1^H, ^13^C NMR spectra and 2D NMR (COSY, ROESY, HSQC and HMBC) performed on the N-methyl derivative unequivocally confirmed the structure of the new diazaphenothiazine system. 10*H*-3-methyl-1,6-diazaphenothiazine was transformed by alkylation reactions into 10-substituted derivatives containing N-methyl, N-allyl, N-propargyl and N-benzyl substituents (**B7**–**B10**). All derivatives were tested against skin cancer cell lines, including melanotic (COLO829, G361) and amelanotic (A375, C32) melanoma cells and normal human fibroblasts (HDF), to determine their IC_50_ values using a WST-1 assay. All tested compounds showed high cytotoxic potential towards the tested melanoma cells (COLO829, G361) and amelanotic melanoma cells (A375, C32), with IC_50_ values ranging from 17 to 137 µM. This activity was dependent on both the tested line and the type of substituent on the nitrogen atom within the dipyridothiazine system. Analysis of cytotoxicity towards normal human fibroblast HDF cells indicated that only compounds **B8** and **B10** containing the appropriate allyl and benzyl substituents showed selectivity and were inactive within the tested concentration range. Moreover, they were much more active against melanotic (COLO829, G361) lines than against amelanotic (A375, C32) melanoma cells. Therefore, we also investigated the effect of these two most promising derivatives on cellular GSH levels in amelanotic melanoma C32 and melanoma G361 cells. The obtained results confirmed a strong ability to induce redox imbalance, which was evidenced by GSH depletion due to excessive ROS production. The ability of the **B8** and **B10** derivatives to induce apoptosis was also tested by analyzing the mitochondrial membrane potential and DNA fragmentation in the tested in vitro experimental models. The results of these studies confirmed that the new dipyridothiazine derivatives increased the number of depolarized (early apoptotic) melanoma cells and have the ability to induce DNA fragmentation, which is characteristic of the late phase of apoptosis. Taking the obtained results into account, it can be considered that the new dipyridothiazines analyzed have a strong ability to initiate apoptosis via the intrinsic signaling pathway and are promising molecules with anticancer potential. However, further studies, with the use of both in vitro (e.g., oxidative stress assessment, selectivity evaluation applying normal human melanocytes) as well as in vivo experimental models, are needed to confirm their potential anti-melanoma activity.

## Figures and Tables

**Table 1:** Calculated concentration corresponding to a 50% cytotoxicity rate (IC_50_) after 24 h (**A**), 48 h (**B**) and 72 h (**C**) of exposure to **B6**–**B10** compounds—the results from the WST-1 assay obtained for amelanotic (A375, C32), melanotic (G361, COLO829) melanoma cells and normal human fibroblasts (HDF) incubated with the compounds in a concentration range of 1–100 µM.

A					
Sample	IC_50_ Values (µM)				
	A375	C32	G361	COLO829	HDF
**B6**	225.6	94.31	134.1	262.2	83.13
**B7**	235.3	91.38	194.1	291.9	55.44
**B8**	392.6	129.1	168.3	220.3	N/D
**B9**	697.8	120.2	116.4	135.6	57.43
**B10**	511.1	95.8	131.7	177.6	1930.0
**B**					
**Sample**	**A375**	**C32**	**G361**	**COLO829**	**HDF**
**B6**	97.85	76.1	56.25	59.04	40.05
**B7**	99.55	89.65	75.37	64.81	31.73
**B8**	165.9	105.3	79.94	64.09	142.3
**B9**	171.2	167.2	97.01	61.45	22.24
**B10**	109.3	87.01	100.2	75.5	931.9
**C**					
**Sample**	**A375**	**C32**	**G361**	**COLO829**	**HDF**
**B6**	39.95	18.14	22.85	30.15	31.95
**B7**	17.64	22.23	41.58	35.46	30.5
**B8**	91.19	59.61	24.57	31.44	125.2
**B9**	137.5	66.41	56.97	49.52	11.31
**B10**	60.39	42.68	43.02	36.96	656.4

N/D—not detectable.

## Data Availability

Data are contained within the article and Supplementary Materials.

## Supplementary Materials

The following supporting information can be downloaded at: https://www.mdpi.com/article/10.3390/molecules30132779/s1. 1H NMR, 13C NMR, HR MS of 3-amino-3′-nitro-5′-methyl-2,2-dipyridinyl sulfide (B3); 1H NMR of 10H-3-methyl-1,6-diazaphenothiazine (B6); 13C NMR of 10H-3-methyl-1,6-diazaphenothiazine (B6); HR MS of 10H-3-methyl-1,6-diazaphenothiazine (B6); 1H NMR of 3,10-dimethyl-1,6-diazaphenothiazine (B7); 13C NMR of 3,10-dimethyl-1,6-diazaphenothiazine (B7); 2D NMR: COSY, ROESY, HSQC, HMBC of 3,10-dimethyl-1,6-diazaphenothiazine (B7); HR MS of 3,10-dimethyl-1,6-diazaphenothiazine (B7); 1H NMR, 13C NMR, HR MS of 3-metyhl-10-allyl-1,6-diazaphenothiazine (B8); 1H NMR, 13C NMR, HR MS of 3-methyl-10-propargyl-3,6-diazaphenothiazines (B9); 1H NMR, 13C NMR, HR MS of 3-methyl-10-benzyl-1,6-diazaphenothiazine (B10).
