# Supplementary material for: Novel 3-Methyl-1,6-Diazaphenothiazine as an Anticancer Agent—Synthesis, Structure, and In Vitro Anticancer Evaluation
Source: Molecules. 2025 Jun 27;30(13):2779. doi: 10.3390/molecules30132779 (PMC12250835; doi:10.3390/molecules30132779)
Supplement: Supplementary file 1 [file molecules-30-02779-s001.zip › molecules-3703540-supplementary.pdf]

Supplementary Material

Novel 3-Methyl-1,6-Diazaphenothiazine as an Anticancer Agent—Synthesis, Structure, and In Vitro Anticancer Evaluation

Beata Morak-Młodawska [1,*], Emilia Martula [1], Małgorzata Jeleń [1], Artur Beberok [2,*], Zuzanna Rzepka [2], Sebastian Musiał [2], Szymon Małek [2], Marta Karkoszka-Stanowska [2] and Dorota Wrześniok [2]

[1] Department of Organic Chemistry, Faculty of Pharmaceutical Sciences in Sosnowiec, Medical University of Silesia, Jagiellońska 4, 41-200 Sosnowiec, Poland
[2] Department of Pharmaceutical Chemistry, Faculty of Pharmaceutical Sciences in Sosnowiec, Medical University of Silesia, Jagiellońska 4, 41-200 Sosnowiec, Poland
* Correspondence: bmlodawska@sum.edu.pl (B.M.-M.); abeberok@sum.edu.pl (A.B.)

Content 1

1. ^{1}H NMR, ^{13}C NMR, HR MS of 3-amino-3`-nitro-5`-methyl-2,2-dipyridinyl sulfide (**B3**) 2
2. ^{1}H NMR of 10H-3-methyl-1,6-diazaphenothiazine (**B6**) 4
3. ^{13}C NMR of 10H-3-methyl-1,6-diazaphenothiazine (**B6**)
4. HR MS of 10H-3-methyl-1,6-diazaphenothiazine (**B6**) 6
5. ^{1}H NMR of 3,10-dimethyl-1,6-diazaphenothiazine (**B7**) 7
6. ^{13}C NMR of 3,10-dimethyl-1,6-diazaphenothiazine (**B7)** 9
7. 2D NMR: COSY, ROESY, HSQC, HMBC of 3,10-dimethyl-1,6-diazaphenothiazine (**B7)** 10
8. HR MS of 3,10-dimethyl-1,6-diazaphenothiazine (**B7)** 14
9. ^{1}H NMR, ^{13}C NMR, HR MS of 3-metyhl-10-allyl-1,6-diazaphenothiazine (**B8**) 15
10. ^{1}H NMR, ^{13}C NMR, HR MS of 3-methyl-10-propargyl-3,6-diazaphenothiazines (**B9**) 18
11. ^{1}H NMR, ^{13}C NMR, HR MS of 3-methyl-10-benzyl-1,6-diazaphenothiazine (**B10**) 21

1. ^{1}H NMR, ^{13}C NMR, HR MS of 3-amino-3`-nitro-5`-methyl-2,2-dipyridinyl sulfide (**B3**)

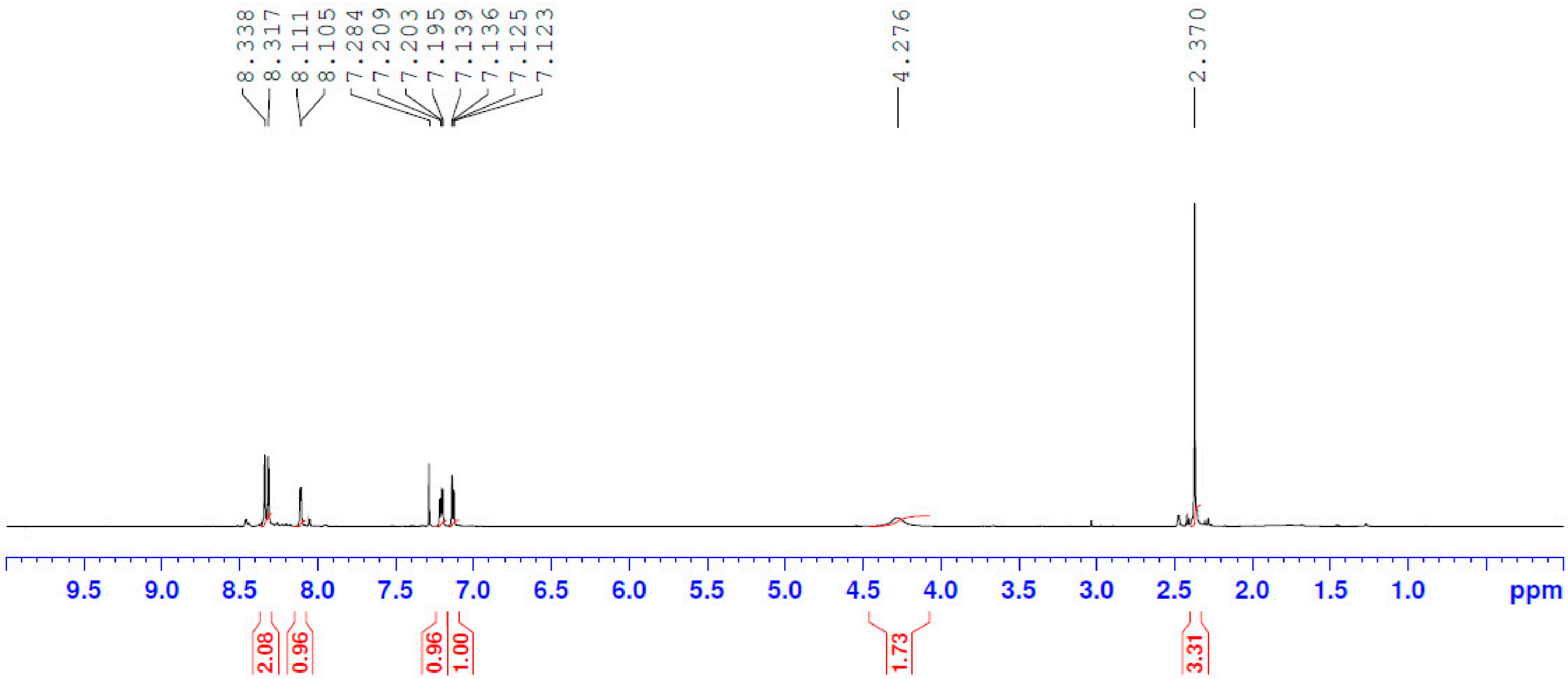

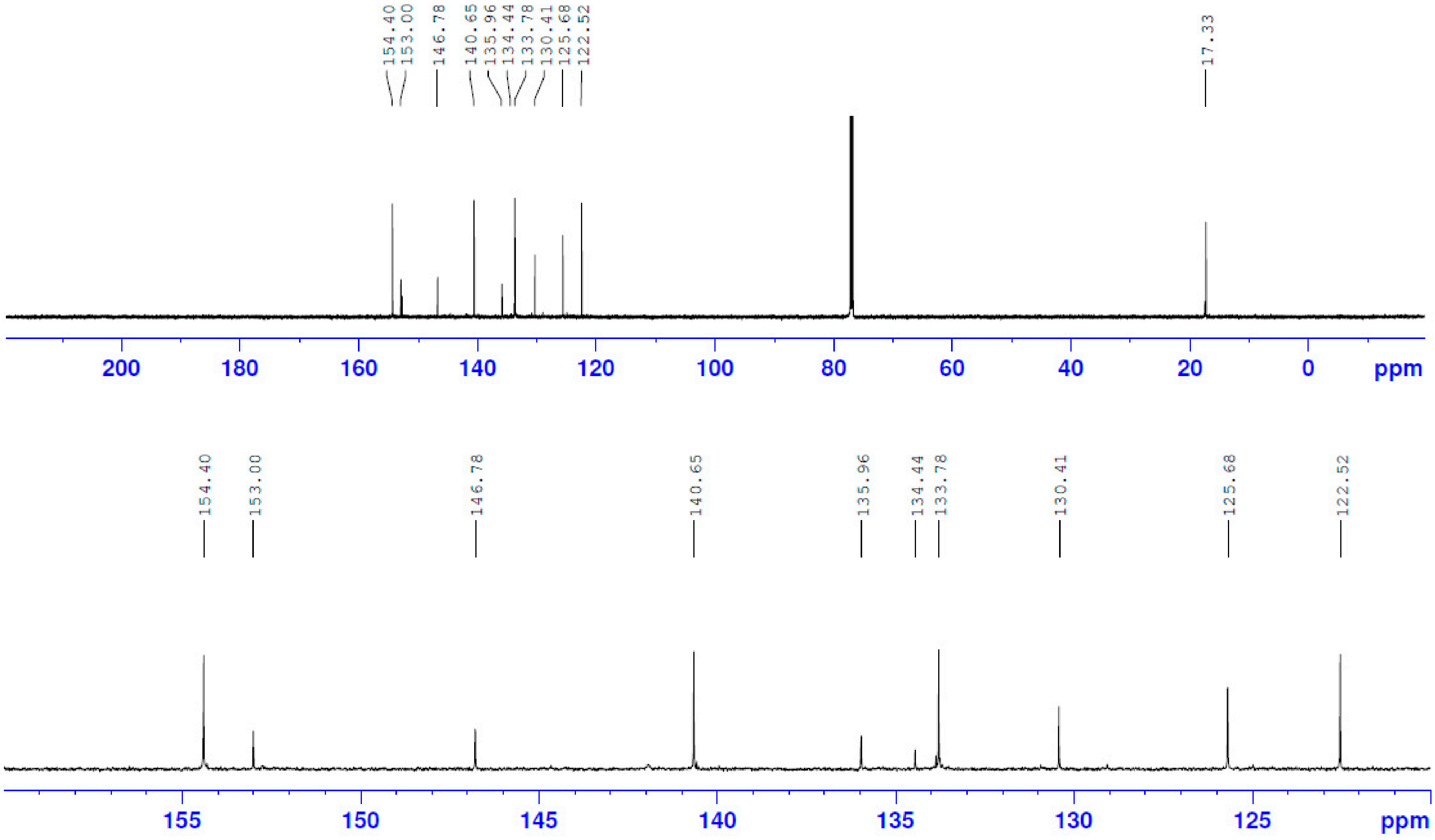
154.40
153.00
146.78
140.65
135.96
134.44
133.78
130.41
125.68
122.52
17.33
200
180
160
140
120
100
80
60
40
20
0
ppm
155
150
145
140
135
130
125
ppm

Acquisition Parameter

Source Type	ESI	Ion Polarity	Positive	Set Nebulizer	0.3 Bar		
Focus	Active	Set Capillary	4000 V	Set Dry Heater	200 °C		
Scan Begin	80 m/z	Set End Plate Offset	-500 V	Set Dry Gas	3.0 l/min		
Scan End	1000 m/z	Set Charging Voltage	2000 V	Set Divert Valve	Source		
		Set Corona	0 nA	Set APCI Heater	0 °C		

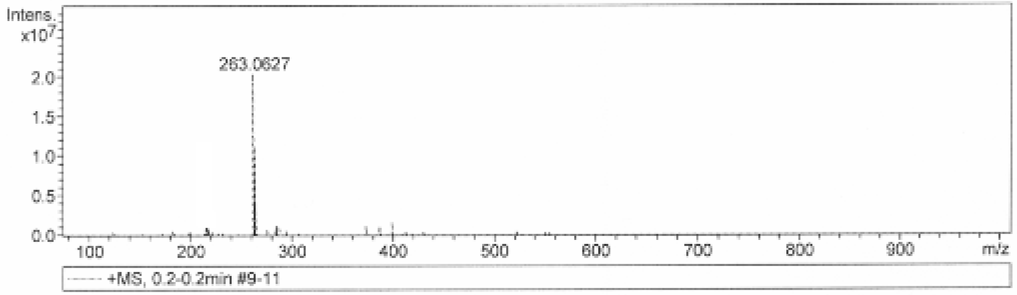

#	m/z	Res.	S/N	I	I %	FWHM
1	263.0627	9254	73182.4	20373500	100.0	0.0284

2. ^{1}H NMR of 10*H*-3-methyl-1,6-diazaphenothiazine (**B6**)

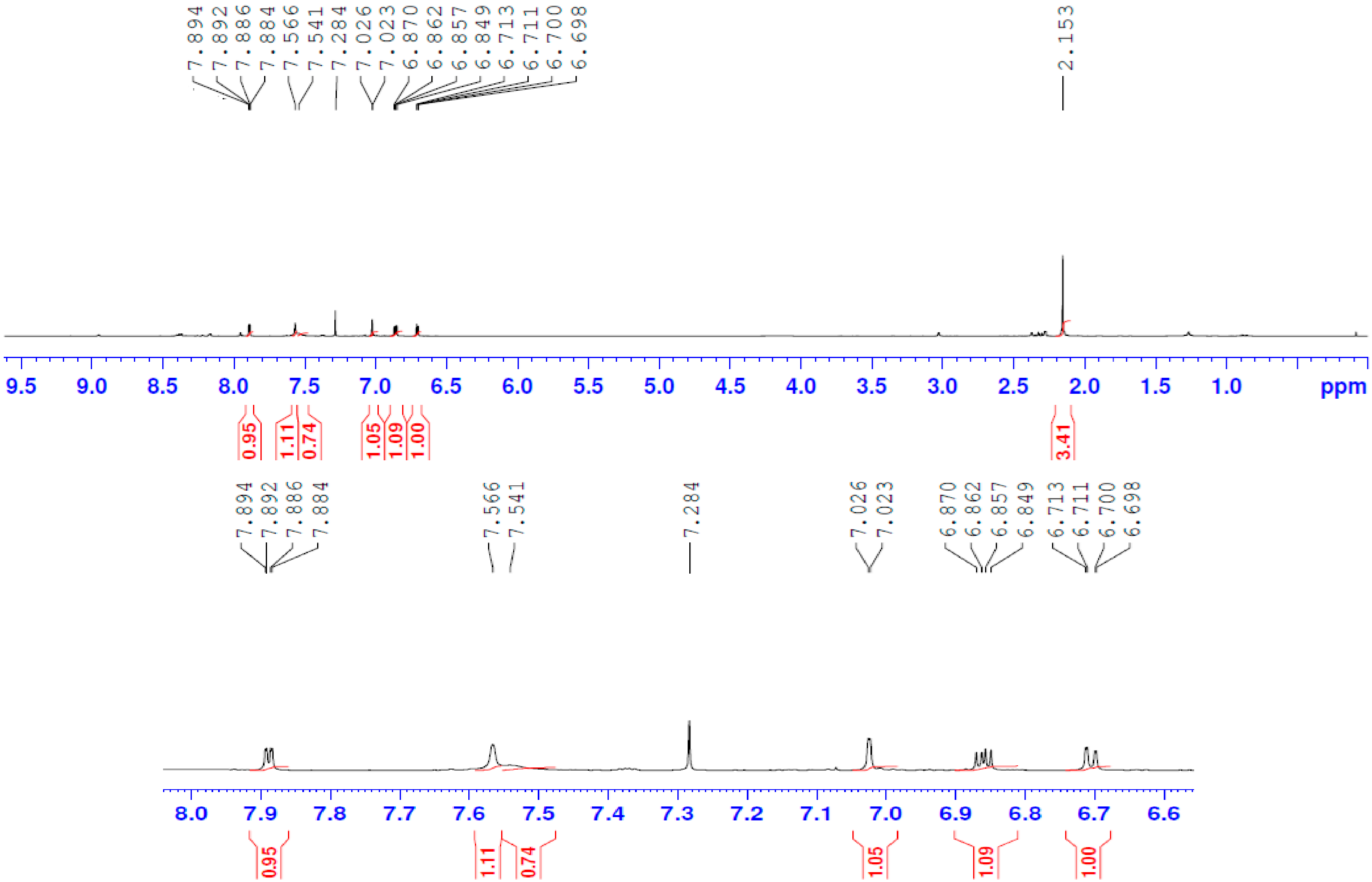

2. ^{13}C NMR of 10*H*-3-methyl-1,6-diazaphenothiazine (**B6**)

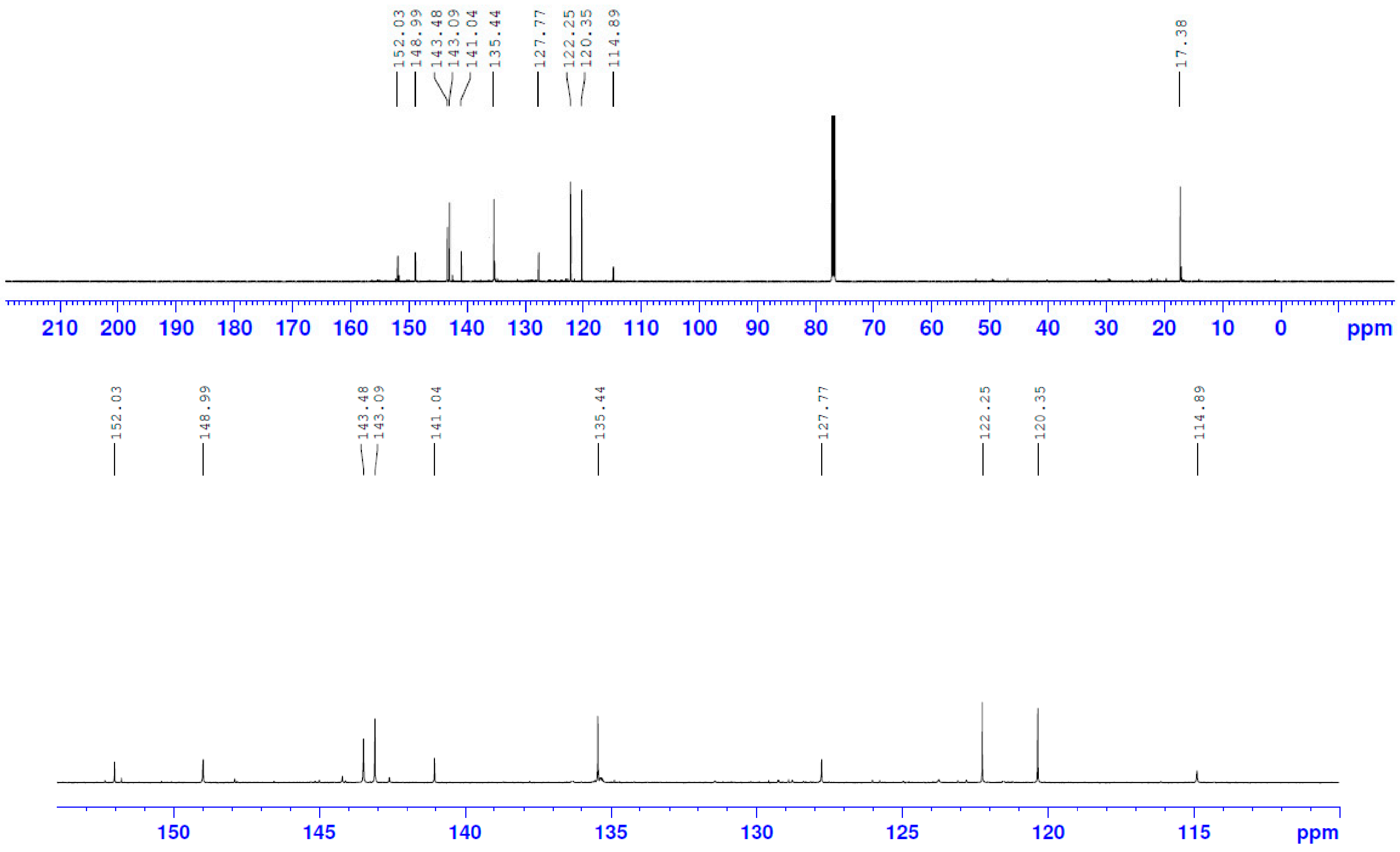

3. HR MS of 10*H*-3-methyl-1,6-diazaphenothiazine (**B6**)

Acquisition Parameter

Source Type	ESI	Ion Polarity	Positive	Set Nebulizer	0.3 Bar
Focus	Active	Set Capillary	4000 V	Set Dry Heater	200 °C
Scan Begin	100 m/z	Set End Plate Offset	-500 V	Set Dry Gas	3.0 l/min
Scan End	1000 m/z	Set Charging Voltage	2000 V	Set Divert Valve	Source
		Set Corona	0 nA	Set APCI Heater	0 °C

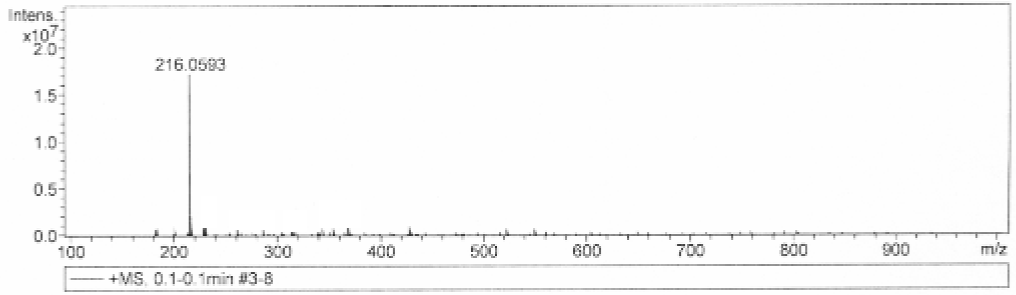

#	m/z	Res.	S/N	I	I %	FWHM
1	216.0593	31734	132736.2	17196490	100.0	0.0068

5. ^{1}H NMR of 3,10-dimethyl-1,6-diazaphenothiazine (**B7**)

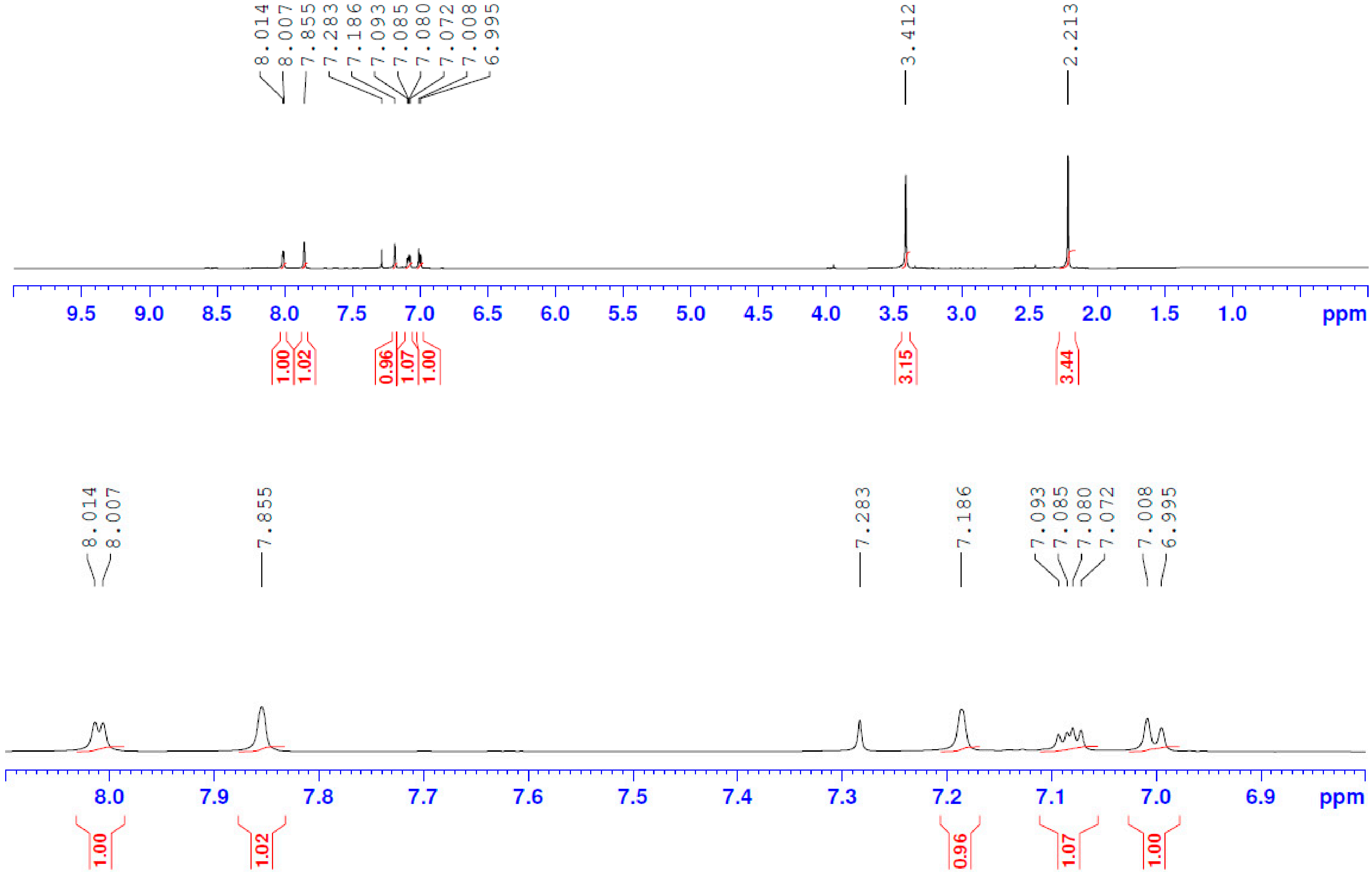

6. ^{13}C NMR of 3,10-dimethyl-1,6-diazaphenothiazine (**B7**)

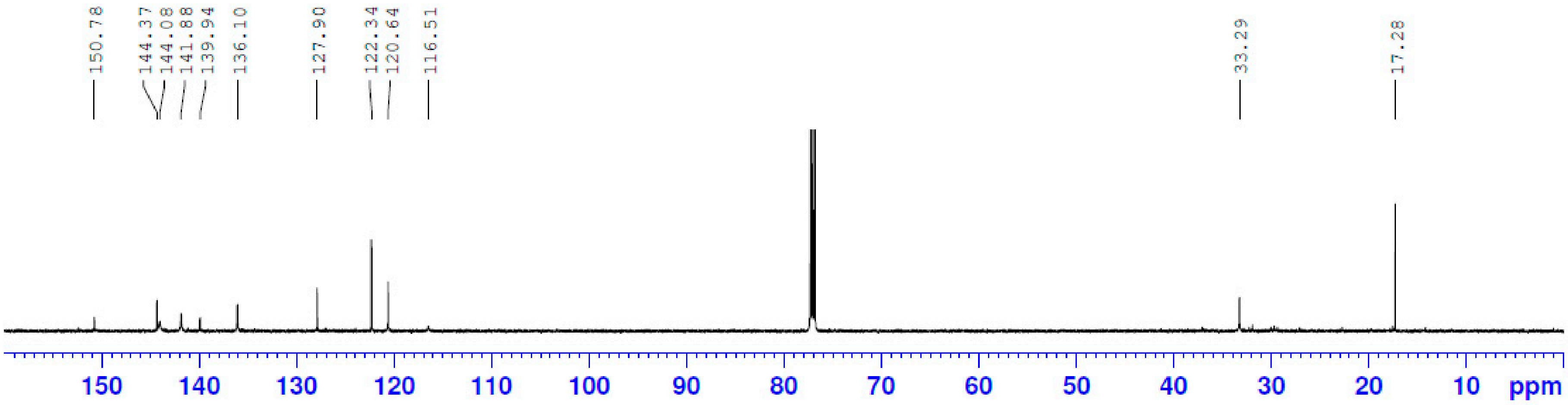

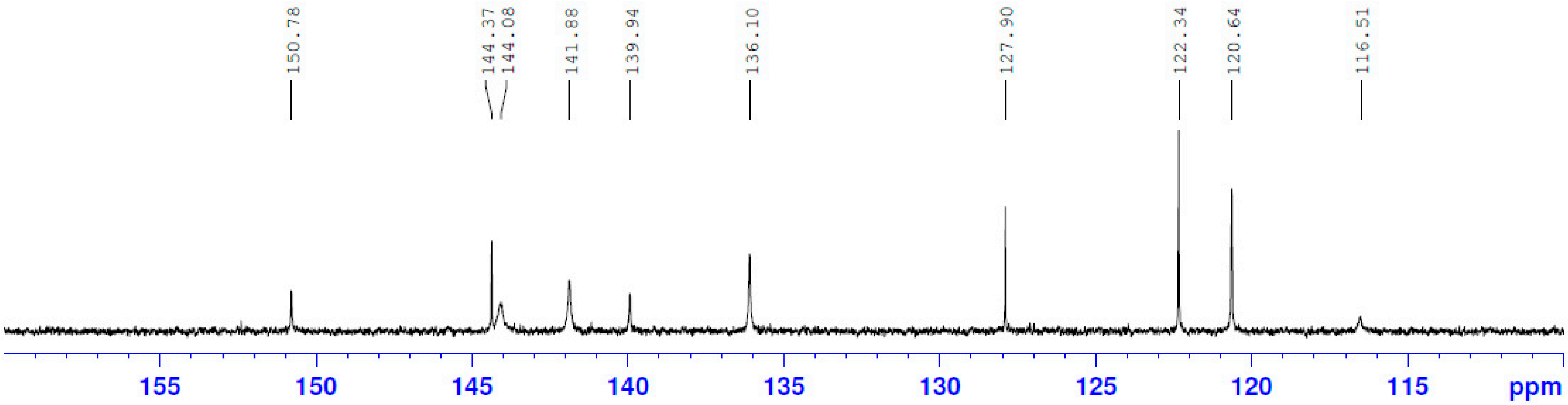

7. 2D NMR: COSY, ROESY, HSQC, HMBC of 3,10-dimethyl-1,6-diazaphenothiazine (**B7**)

a. ^{1}H-^{1}H COSY

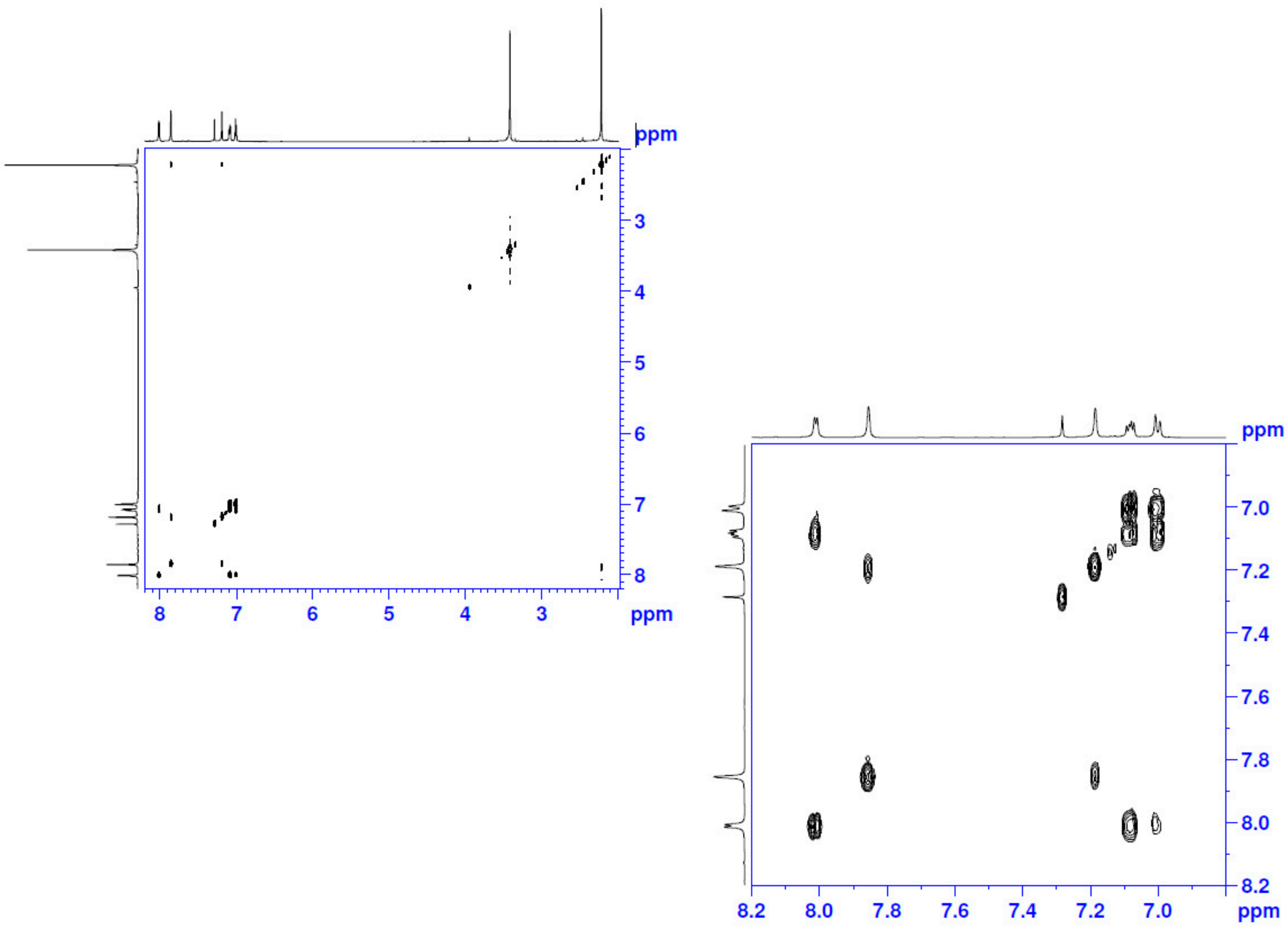

b. ROESY

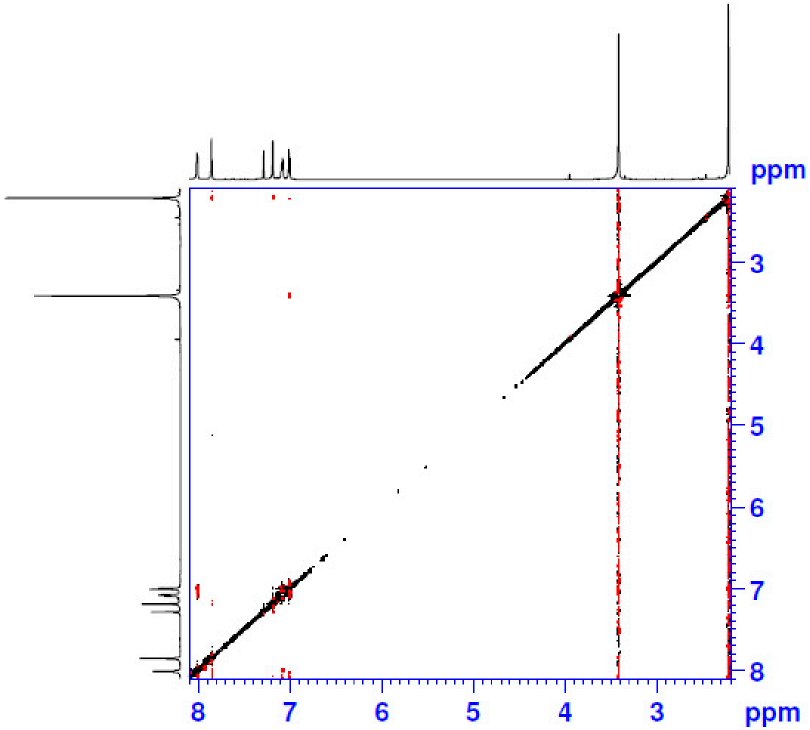

ppm
3
4
5
6
7
8
8
7
6
5
4
3
ppm

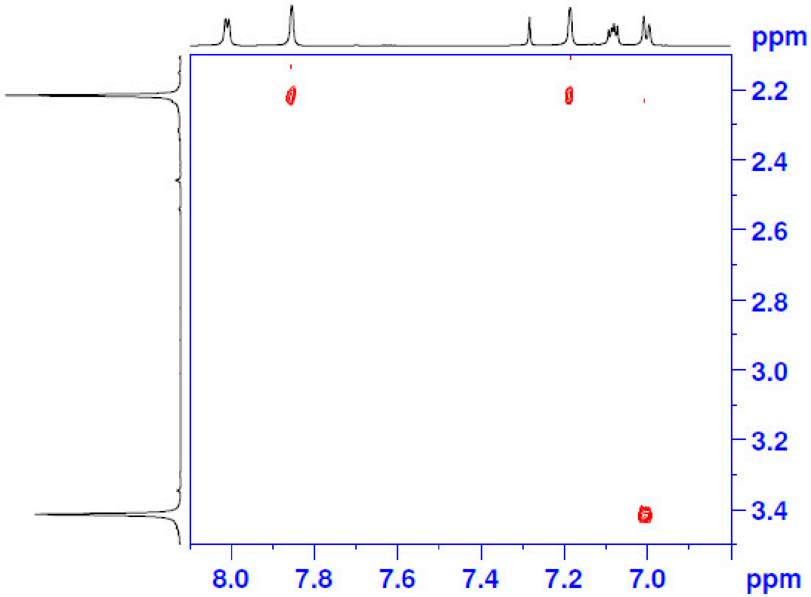

ppm
2.2
2.4
2.6
2.8
3.0
3.2
3.4
8.0
7.8
7.6
7.4
7.2
7.0
ppm

c. HSQC

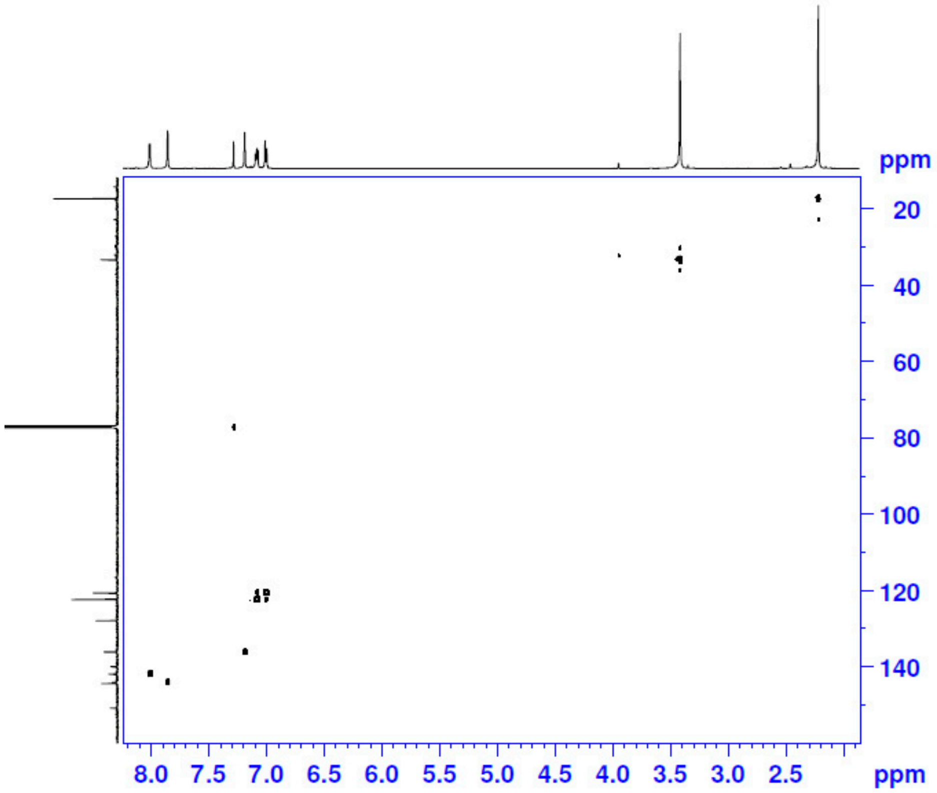
ppm
20
40
60
80
100
120
140
8.0 7.5 7.0 6.5 6.0 5.5 5.0 4.5 4.0 3.5 3.0 2.5
ppm

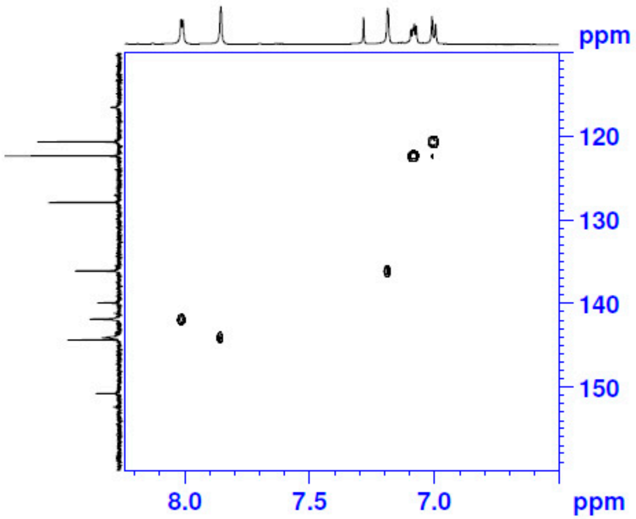
ppm
120
130
140
150
8.0 7.5 7.0
ppm

d. HMBC

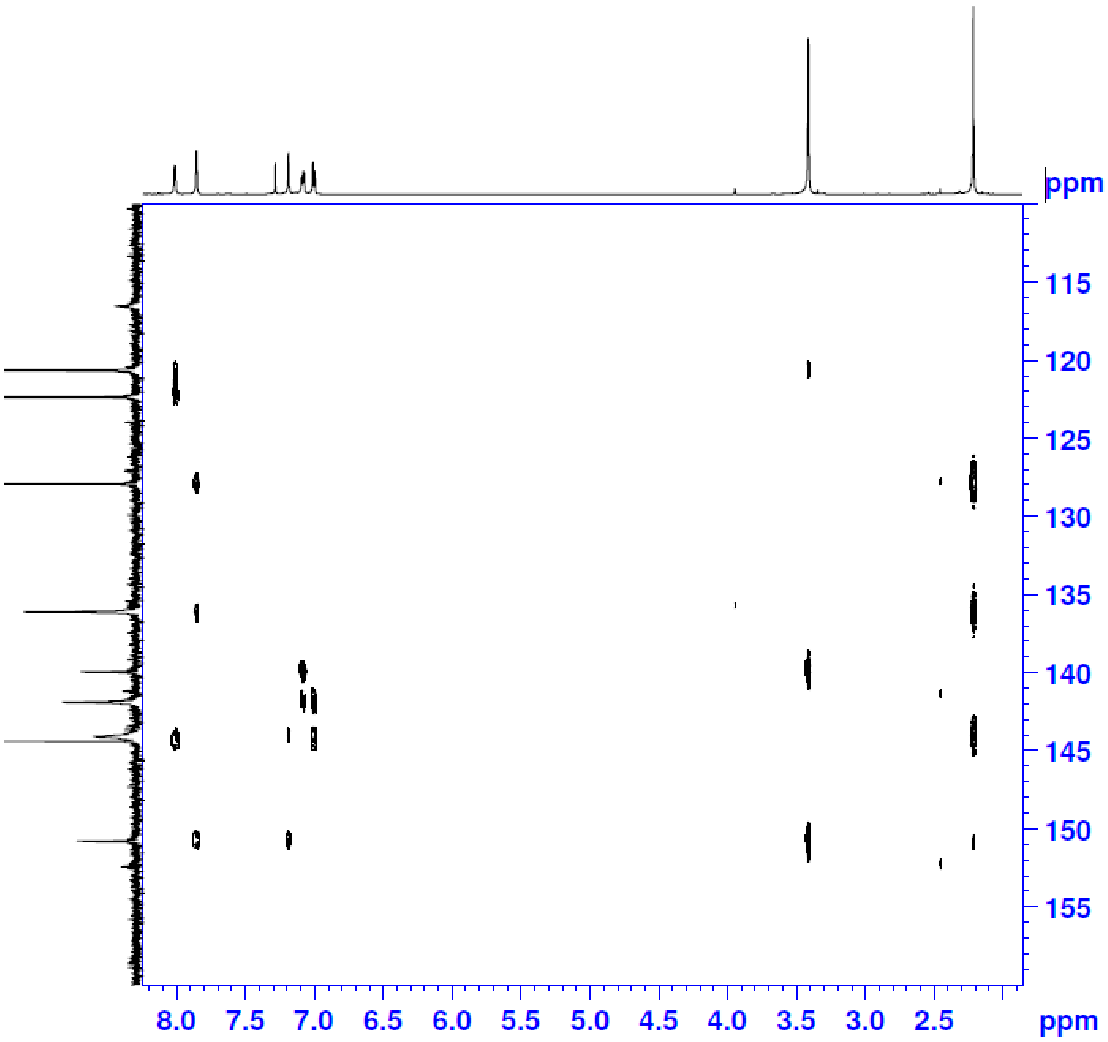
ppm
115
120
125
130
135
140
145
150
155
8.0 7.5 7.0 6.5 6.0 5.5 5.0 4.5 4.0 3.5 3.0 2.5
ppm

10. HR MS of 3,10-dimethyl-1,6-diazaphenothiazine (**B7**)

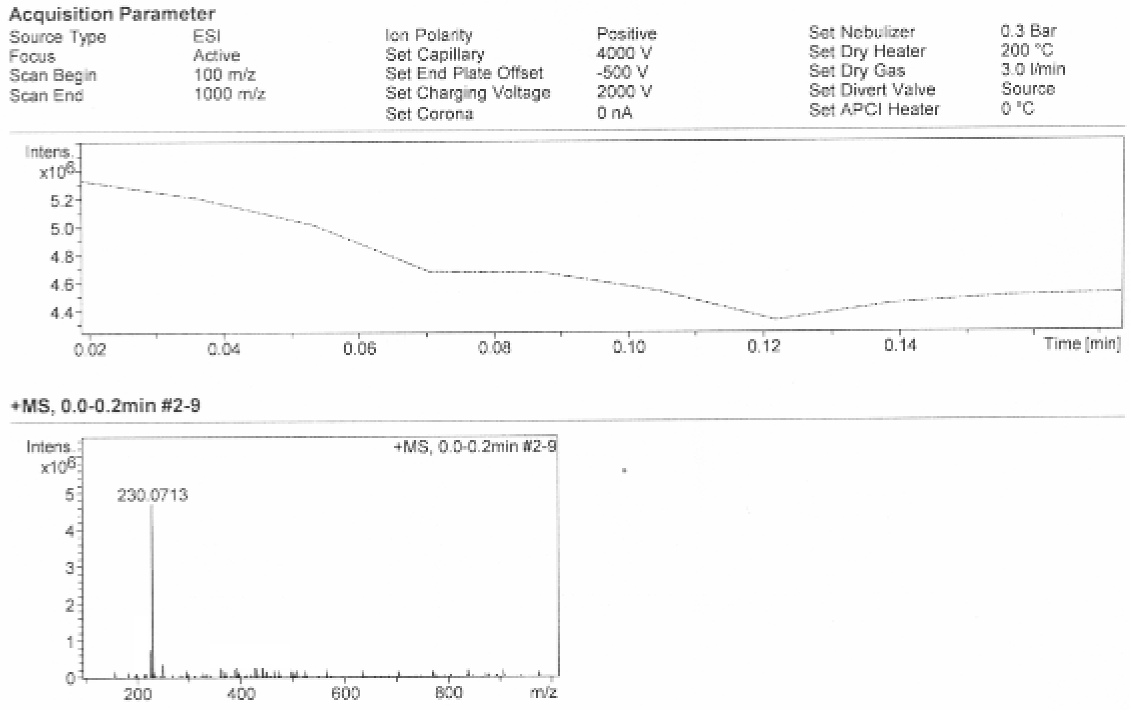

^{1}H NMR, ^{13}C NMR, HR MS of 3-metyhl-10-allyl-1,6-diazaphenothiazine (**B8**)

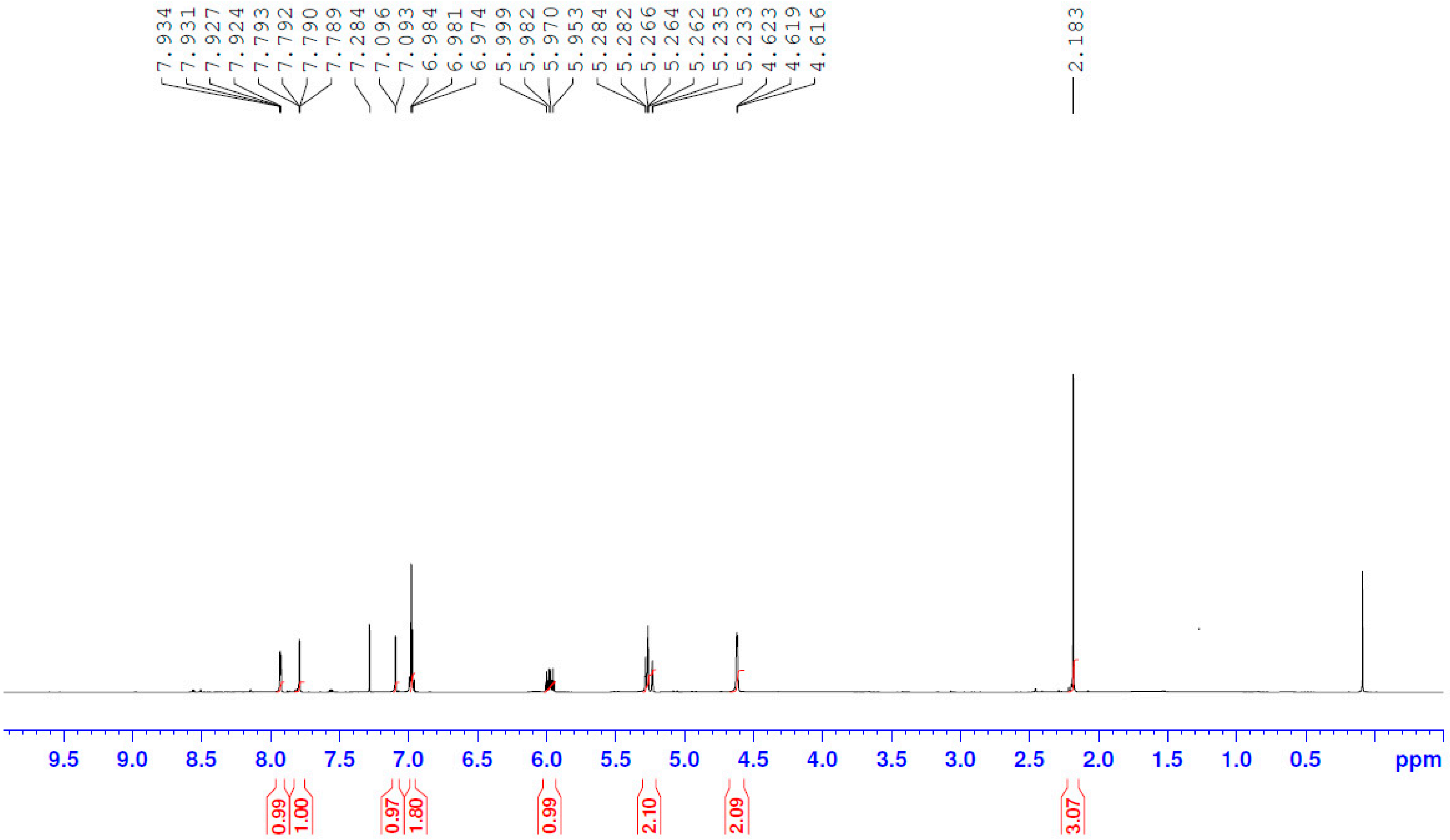

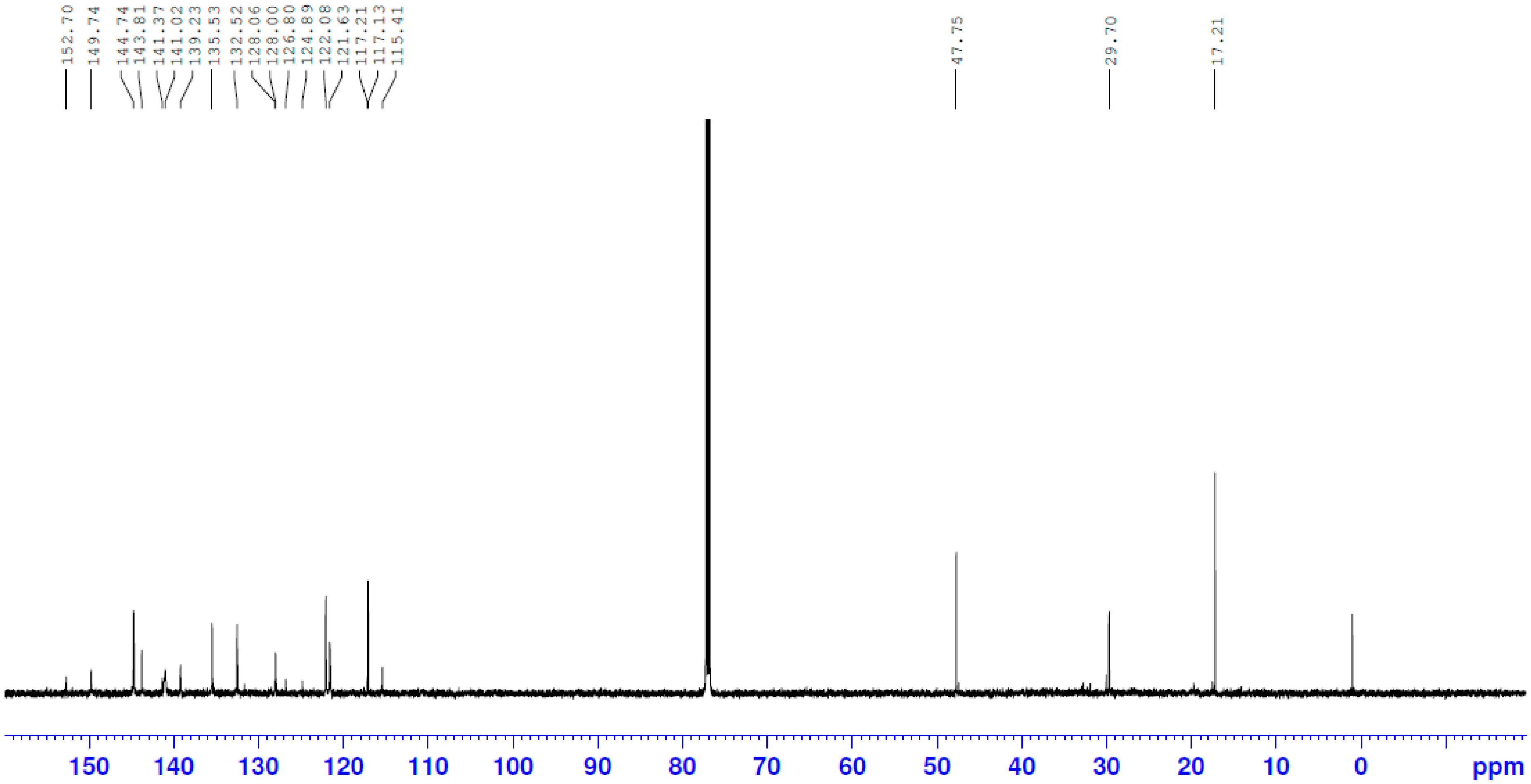
152.70
149.74
144.74
143.81
141.37
141.02
139.23
135.53
132.52
128.06
128.00
126.80
124.89
122.08
121.63
117.21
117.13
115.41
47.75
29.70
17.21
150
140
130
120
110
100
90
80
70
60
50
40
30
20
10
0
ppm

Acquisition Parameter

Source Type	APCI	Ion Polarity	Positive	Set Nebulizer	2.0 Bar
Focus	Active	Set Capillary	4000 V	Set Dry Heater	200 °C
Scan Begin	100 m/z	Set End Plate Offset	-500 V	Set Dry Gas	4.0 l/min
Scan End	2200 m/z	Set Charging Voltage	2000 V	Set Divert Valve	Source
		Set Corona	4000 nA	Set APCI Heater	450 °C

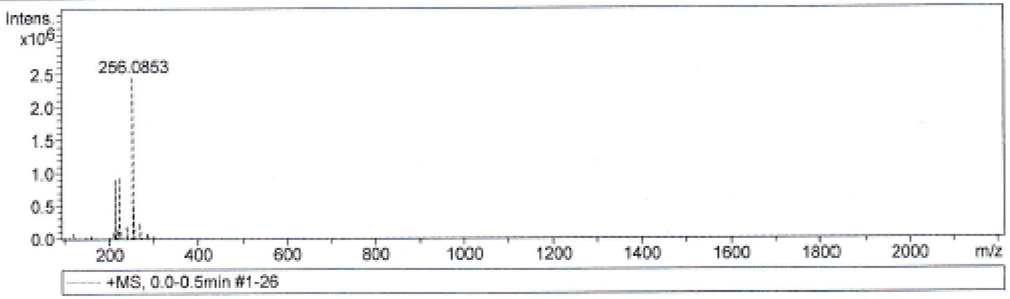

#	m/z	Res.	S/N	I	I %	FWHM
1	215.0465	30524	29409.6	928261	37.8	0.0070
2	225.1914	29798	26657.5	892211	36.3	0.0076
3	256.0853	29111	60794.2	2456563	100.0	0.0088

^{1}H NMR, ^{13}C NMR, HR MS of 3-methyl-10-propargyl-3,6-diazaphenothiazines (**B9**)

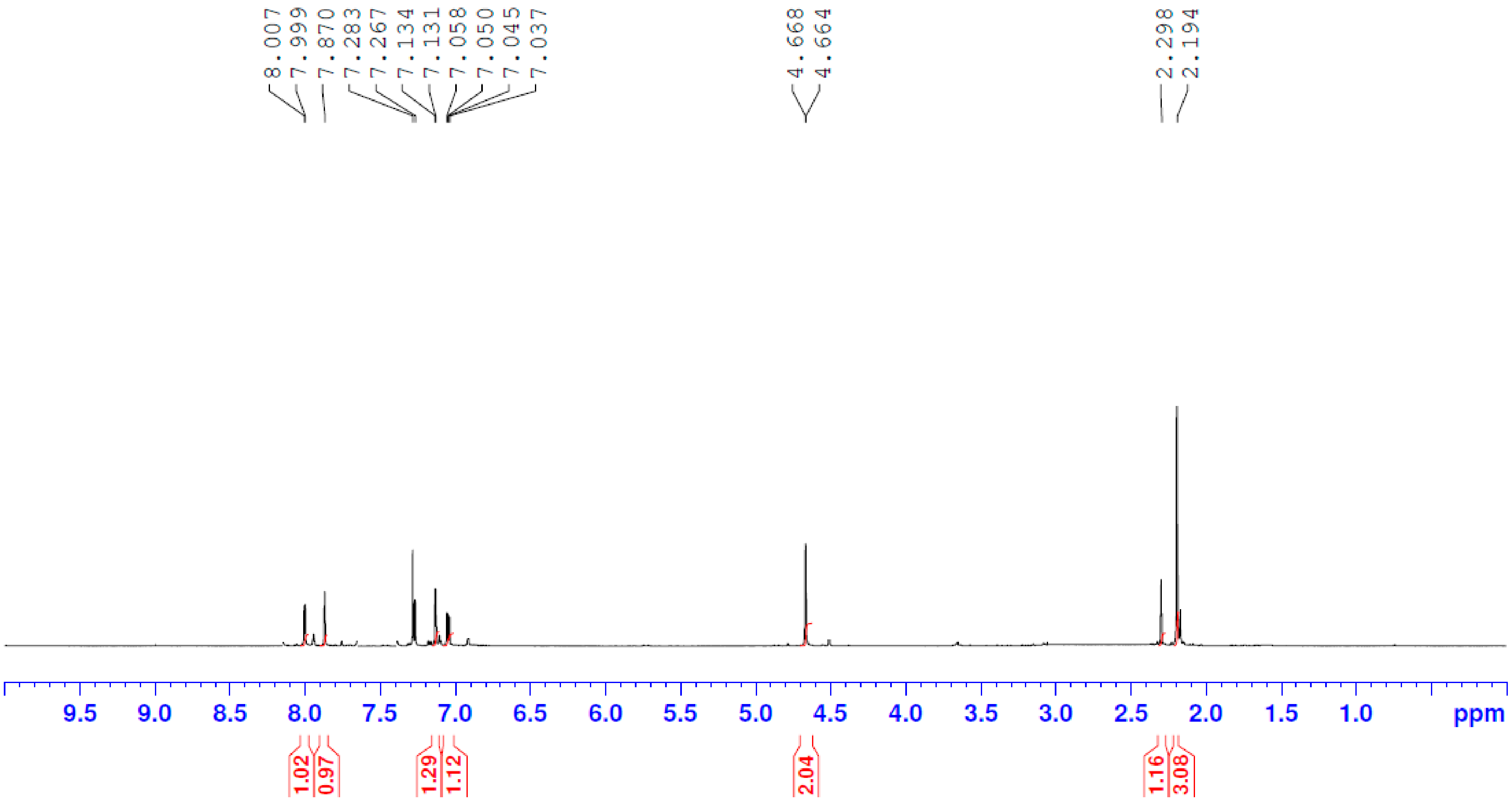

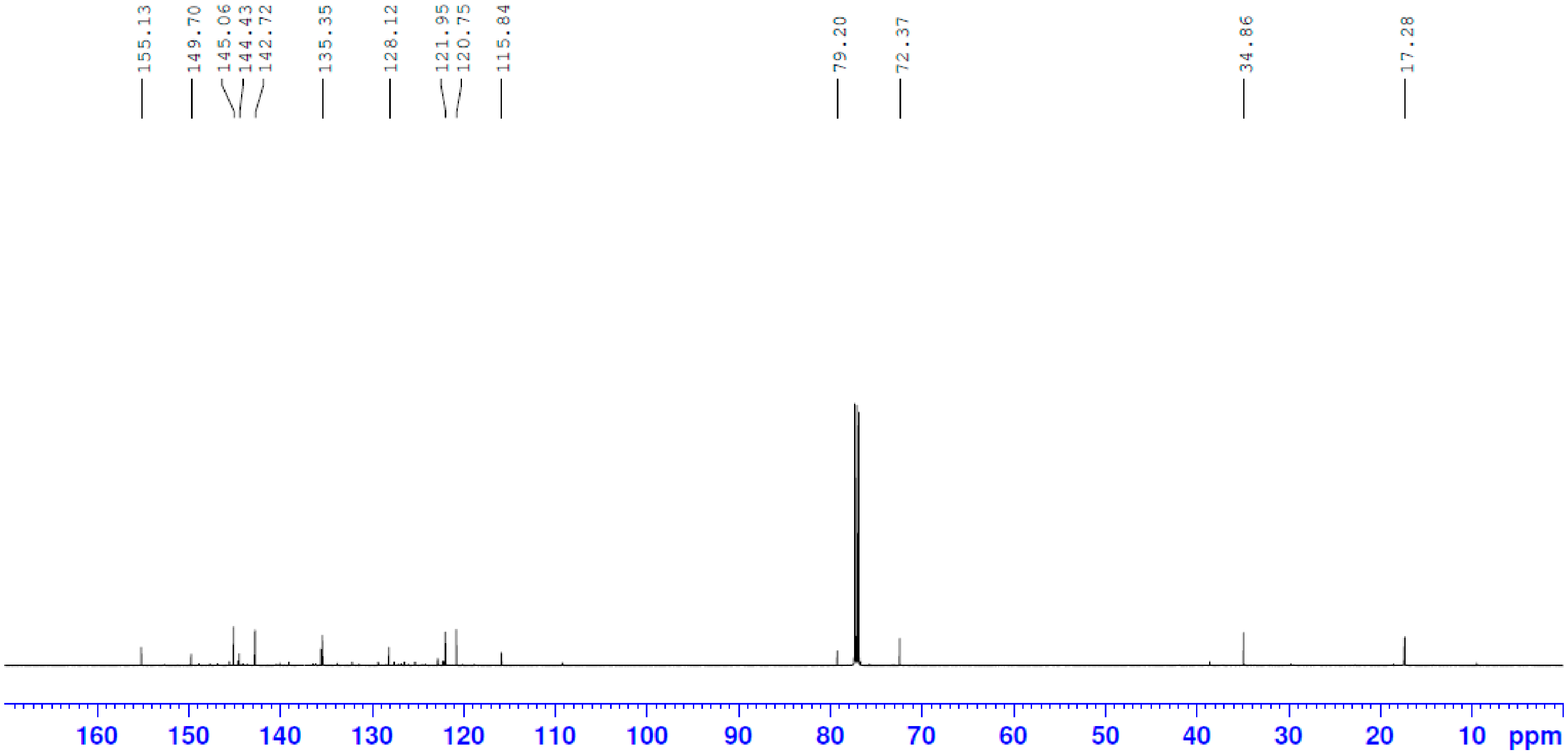
155.13
149.70
145.06
144.43
142.72
135.35
128.12
121.95
120.75
115.84
79.20
72.37
34.86
17.28
160
150
140
130
120
110
100
90
80
70
60
50
40
30
20
10
ppm

Acquisition Parameter

Source Type	ESI	Ion Polarity	Positive	Set Nebulizer	0.3 Bar
Focus	Active	Set Capillary	4000 V	Set Dry Heater	200 °C
Scan Begin	100 m/z	Set End Plate Offset	-500 V	Set Dry Gas	3.0 l/min
Scan End	1000 m/z	Set Charging Voltage	2000 V	Set Divert Valve	Source
		Set Corona	0 nA	Set APCI Heater	0 °C

+MS, 0.0-0.4min #1-23

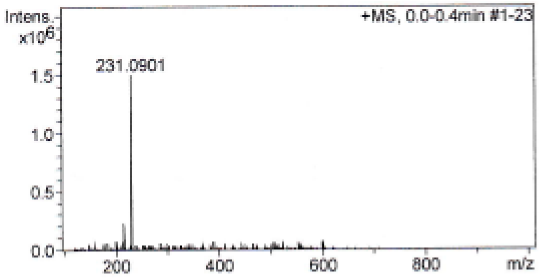

#	m/z	Res.	S/N	I	I %	FWHM
1	231.0901	23548	10095.3	1492885	100.0	0.0098

^{1}H NMR, ^{13}C NMR, HR MS of 3-methyl-10-benzyl-1,6-diazaphenothiazine (**B10**)

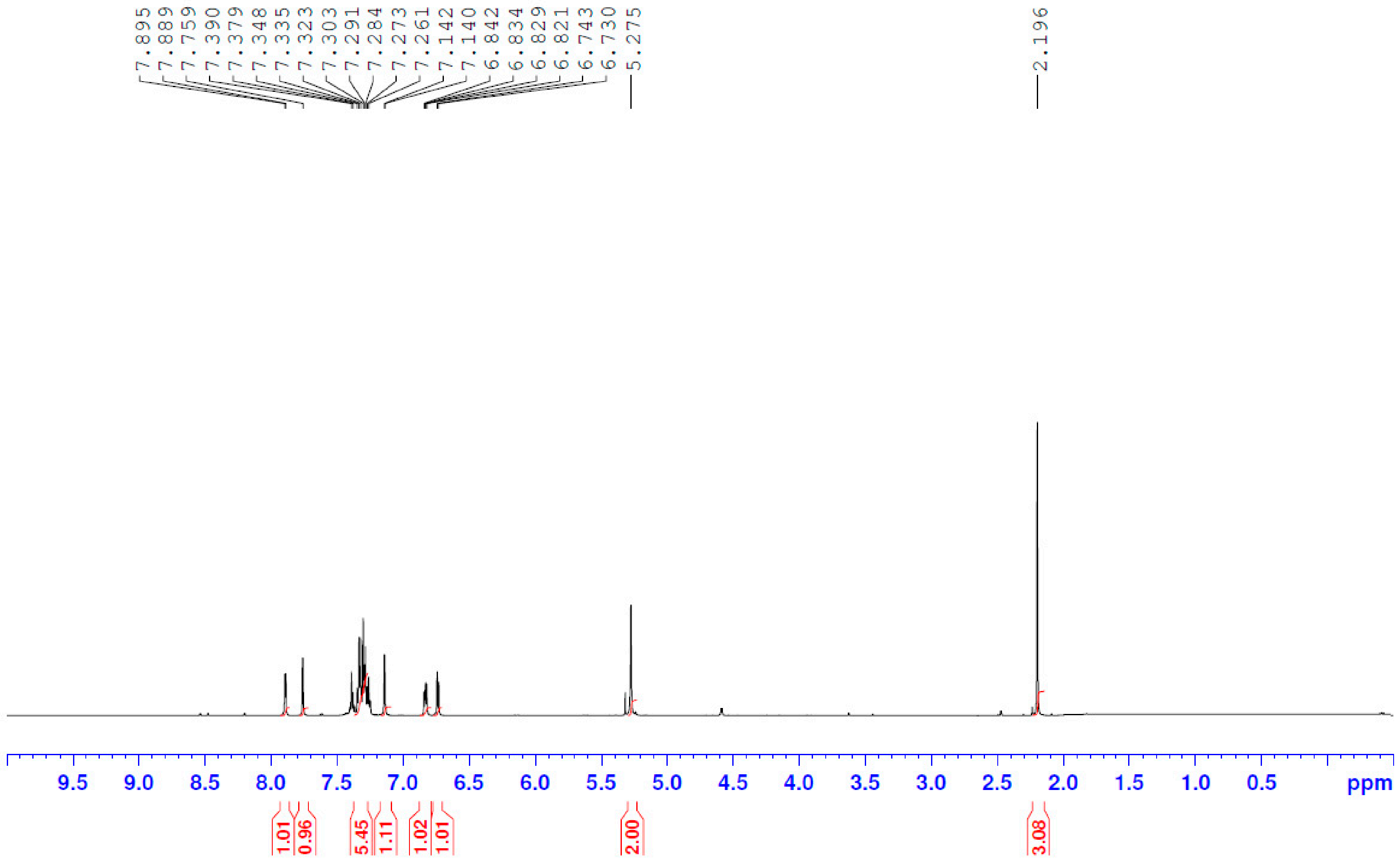

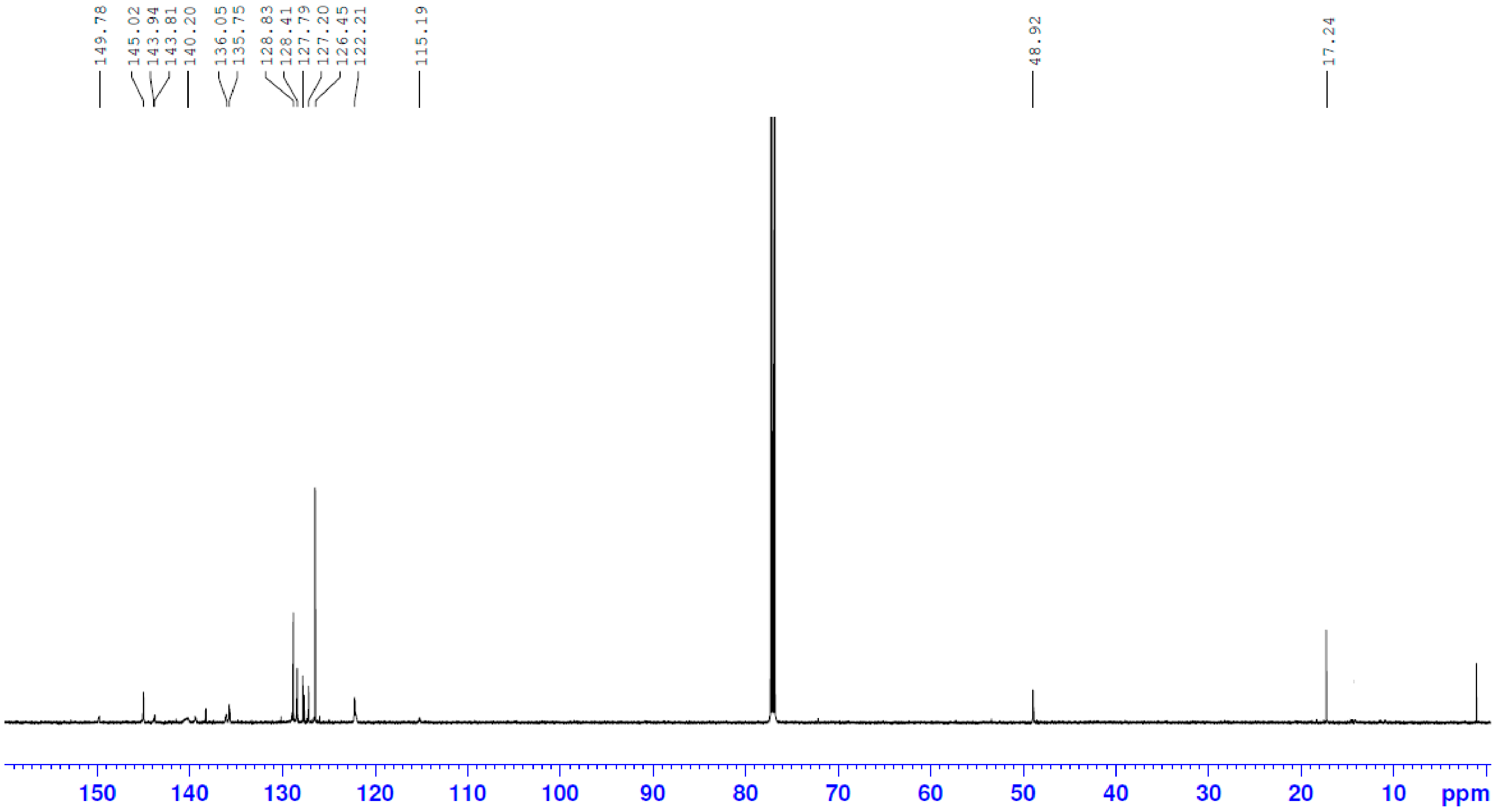
149.78
145.02
143.94
143.81
140.20
136.05
135.75
128.83
128.41
127.79
127.20
126.45
122.21
115.19
48.92
17.24
150 140 130 120 110 100 90 80 70 60 50 40 30 20 10 ppm

Acquisition Parameter

Source Type	ESI	Ion Polarity	Positive	Set Nebulizer	0.3 Bar
Focus	Active	Set Capillary	4000 V	Set Dry Heater	200 °C
Scan Begin	80 m/z	Set End Plate Offset	-500 V	Set Dry Gas	3.0 l/min
Scan End	1000 m/z	Set Charging Voltage	2000 V	Set Divert Valve	Source
		Set Corona	0 nA	Set APCI Heater	0 °C

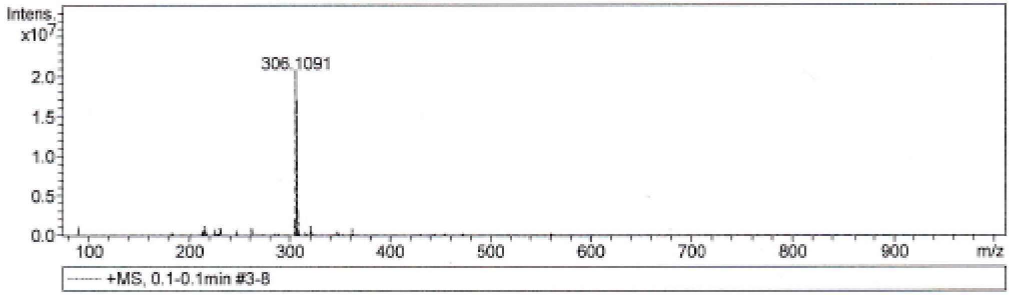

#	m/z	Res.	S/N	I	I %	FWHM
1	306.1091	9846	66586.0	20373500	100.0	0.0311
